# Maternal antibiotic exposure-mediated alterations in basal, and allergen-induced lung function are associated with altered recruitment of eosinophils to the developing lung

**DOI:** 10.3389/fimmu.2025.1715675

**Published:** 2025-12-18

**Authors:** Adrienne N Wilburn, Rabia Ülkü Korkmaz, Jaclyn W McAlees, Julie M Hargis, Schmaiel Shirdel, Imke Lingel, Miki Watanabe-Chailland, Lindsey Romick-Rosendale, Inken Schmudde, James P Bridges, Claire A Chougnet, Hitesh Deshmukh, William J Zacharias, Jörg Köhl, Peter Konig, Yves Laumonnier, Marc Rothenberg, David B Haslam, Ian P Lewkowich

**Affiliations:** 1 Immunology Graduate Program, University of Cincinnati, Cincinnati, OH, United States; 2 Division of Immunobiology, Cincinnati Children’s Hospital Medical Center, Cincinnati, OH, United States; 3 Helmholtz Zentrum München, German Research Center for Environmental Health, Institute of Asthma and Allergy Prevention, Neuherberg, Germany; 4 Airway Research Center North (ARCN), Member of the German Center for Lung Research (DZL), Lübeck, Germany; 5 Institute of Anatomy, University of Lübeck, Lübeck, Germany; 6 Translational Metabolomics Facility, Division of Pathology and Laboratory Medicine, Cincinnati Children’s Hospital Medical Center, Cincinnati, OH, United States; 7 Institute of Nutritional Medicine, University of Lübeck, Lübeck, Germany; 8 Division of Pulmonary, Critical Care and Sleep Medicine, National Jewish Hospital, Denver, CO, United States; 9 Division of Pulmonary Sciences and Critical Care Medicine, University of Colorado Anschutz Medical Campus, Denver, CO, United States; 10 Department of Pediatrics, University of Cincinnati, Cincinnati, OH, United States; 11 Division of Neonatology and Pulmonary Biology, Cincinnati Children’s Hospital Medical Center, Cincinnati, OH, United States; 12 Institute for Systemic Inflammation Research, University of Lübeck, Lübeck, Germany; 13 Division of Allergy and Immunology, Cincinnati Children’s Hospital Medical Center, Cincinnati, OH, United States; 14 Division of Infectious Diseases, Cincinnati Children’s Hospital Medical Center, Cincinnati, OH, United States

**Keywords:** asthma, eosinophil (EOS), lung development, microbiome, allergic disease

## Abstract

**Introduction:**

Early-life dysbiosis is associated with increased risk of asthma development but the underlying mechanisms remain unclear. Although eosinophils have been reported in the developing lung, their contributions to alveolar morphogenesis and lung mechanics have not been functionally interrogated.

**Methods:**

Maternal exposure to antibiotics (ABX) was used to induce early-life offspring dysbiosis, and the effects on lung function and development was assessed. Similar measurements were made in mice lacking eosinophils due to genetic modification, or administration of IL-5 blocking agents.

**Results:**

ABX exposure between Embryonic Day 15 (E15) and post-natal day 28 (PN28), increased allergen-induced, and baseline airway hyperreactivity (AHR). Similar observations were made when maternal ABX exposure was limited to PN10 to PN20. Complete characterization of baseline lung mechanics demonstrated downward-shifted pulmonary PV loops, increased small airway resistance, decreased compliance, and reduced inspiratory capacity at weaning and 14 months of age. Consistent with observation of small airway dysfunction, offspring of ABX-exposed dams demonstrated significantly smaller alveoli at multiple stages of lung development. Examination of recruitment to developing lungs demonstrated an exaggerated recruitment of eosinophils at key developmental periods (PN14) in offspring of ABX-exposed dams. Mice with fewer eosinophils (through genetic knockout, or treatment with anti-IL-5) display altered patterns of lung mechanics opposite to that seen in offspring of ABX-exposed dams.

**Discussion:**

These data underscore an underappreciated role of eosinophils in homeostatic lung development and suggest that early life modulation of pulmonary eosinophil activity has long-term effects on susceptibility to the development of chronic lung diseases such as asthma.

## Introduction

The prevalence of asthma has been increasing in recent decades; in the US, the prevalence of asthma is currently 8.7% (1). Although genetics clearly contributes to the overall risk of developing asthma (2), the recent rises in asthma prevalence are inconsistent with a purely genetic etiology. This has led to speculation that exposure to changing environmental factors, particularly in early life, when important developmental processes are ongoing, synergize with genetic susceptibility, to contribute to the observed rise in asthma prevalence (3). The Developmental Origins of Health and Disease (DOHaD) hypothesis posits that environmental exposures that alter homeostatic developmental trajectories have life-long influences on susceptibility to the development of chronic diseases (4, 5). Consistent with this hypothesis, recent evidence suggests that limiting or altering the types of microbes persistently present at mucosal surfaces (i.e. inducing dysbiosis) in early life is an important risk factor for subsequent asthma development (6–11). However, while the associations between early life dysbiosis and asthma development are strong, the precise mechanisms linking these effects are unclear.

Lung development is a complex process that proceeds sequentially through multiple stages including embryonic, pseudoglandular, canalicular, saccular and alveolarization stages (12, 13). While the majority of lung development occurs prenatally, in both humans and mice, alveolarization occurs postnatally. This final stage involves formation of the gas exchange units of the lung through thinning of the mesenchyme, formation of primary and secondary septae, and close apposition of the capillary bed with alveolar type I epithelial cells. In humans the alveolarization process extends for several years, with the complete complement of alveoli being established between 3 and 15 years of life (14). The final stages of lung development, particularly secondary septation, seem to be associated with robust, and temporally limited recruitment of cells typically associated with Th2 responses – type 2 innate lymphoid cells, and eosinophils (15–19). However, the precise role that eosinophils and other type 2 associated cells play in the regulation of lung development, and the signals that recruit them into the developing lung, remain underdefined.

Although our work, and that of others (20–41) have linked antibiotic exposure to worse asthma outcomes in mouse models, here, we extend these observations, demonstrating that maternal antibiotic exposure during windows of homeostatic post-natal lung development 1) drives transient dysbiosis in offspring; 2) is associated with significant changes in the trajectory of lung development and baseline lung mechanics that are durable, being evident in both neonatal and aged mice (>14 months of age) and 3) increases recruitment of eosinophils to the lung during peak periods of neonatal homeostatic lung remodeling. Reducing pulmonary eosinophil recruitment during these windows through genetic targeting, or application of anti-IL-5, impacts lung mechanics with a directionality that is opposite to that observed in offspring of ABX-exposed dams, Collectively, these data suggest that eosinophils play an underappreciated role in the regulation of lung development, and that altering the recruitment of eosinophils to the developing lung (either augmenting eosinophil recruitment through maternal antibiotic exposure, or limiting eosinophil recruitment through application of IL-5-blocking reagents), directly influences the trajectory of lung development, and alters baseline lung mechanics. As changes in the trajectory of lung development (42–44), and increased baseline airway reactivity (as evidenced by increased “wheeziness” in infancy) (45–48) are associated with increased risk of asthma development, this may provide a mechanistic explanation for how exposures in early life can have a long-lasting influence on asthma susceptibility.

## Materials and methods

### Mice and antibiotics exposure

Animal studies were approved by Cincinnati Children’s Hospital IACUC. Male and female BALB/c mice, eosinophil deficient mice (ΔdblGATA) on a BALB/c background were bred in house. C57BL/6 mice were purchased from Jackson Laboratories. All mice were maintained in a specific pathogen-free facility. Where indicated drinking water of dams was supplemented with sucralose (0.5mg/ml) +/- antibiotics (1mg/ml; vancomycin, ampicillin, and gentamicin; GoldBio) for the indicated periods. Sucralose was added to mitigate the bitter antibiotics flavor and encourage water consumption. Water was replaced with fresh supplemented water every 4–6 days. At the conclusion of the period of antibiotics, dams were provided regular drinking water. Pups were weaned at PN28.

### Flow cytometric analysis of pulmonary cell numbers

Lung tissue was collected, minced in digestion media (RPMI 1640 supplemented with Pen Strep Glutamine (1x; Gibco), liberase [1.5 mg/ml, Roche, Mannheim, Germany], DNase I (0.5 mg/ml, Sigma-Aldrich)) then incubated at 37°C for 45min. Cells were strained through a 70 µm filter and incubated with ACK lysis buffer (Gibco) to remove RBCs. After inactivation and removal of ACK, cells were stained for flow cytometry studies. Cells from lung single cell suspensions were plated at 1,000,000 cells per well. Fc receptors were blocked by incubating cells in supernatant from an anti-mouse CD16/32 producing hybridoma (clone 2.4G2) for 30 minutes at 37 degrees. Cells were stained with the APC-conjugated anti-mouse CD11c (clone N418 (eBioscience), 1:800 dilution), anti-mouse Siglec F (clone E50-2440 (BD Bioscience), 1:300 dilution), BV711-conjugated anti-mouse CD125 (clone T21 (BD Biosciences) 1:400 dilution), and PE-conjugated anti-mouse CD101 (clone Moushi (eBioscience), 1:600 dilution). Samples were acquired on a BD LSRFortessa with lasers tuned to 355 nm, 405 nm, 488 nm, 561 nm, and 635 nm. Flow cytometric data was analyzed with FlowJo.

### Treatment of mice

**Figure 1:**
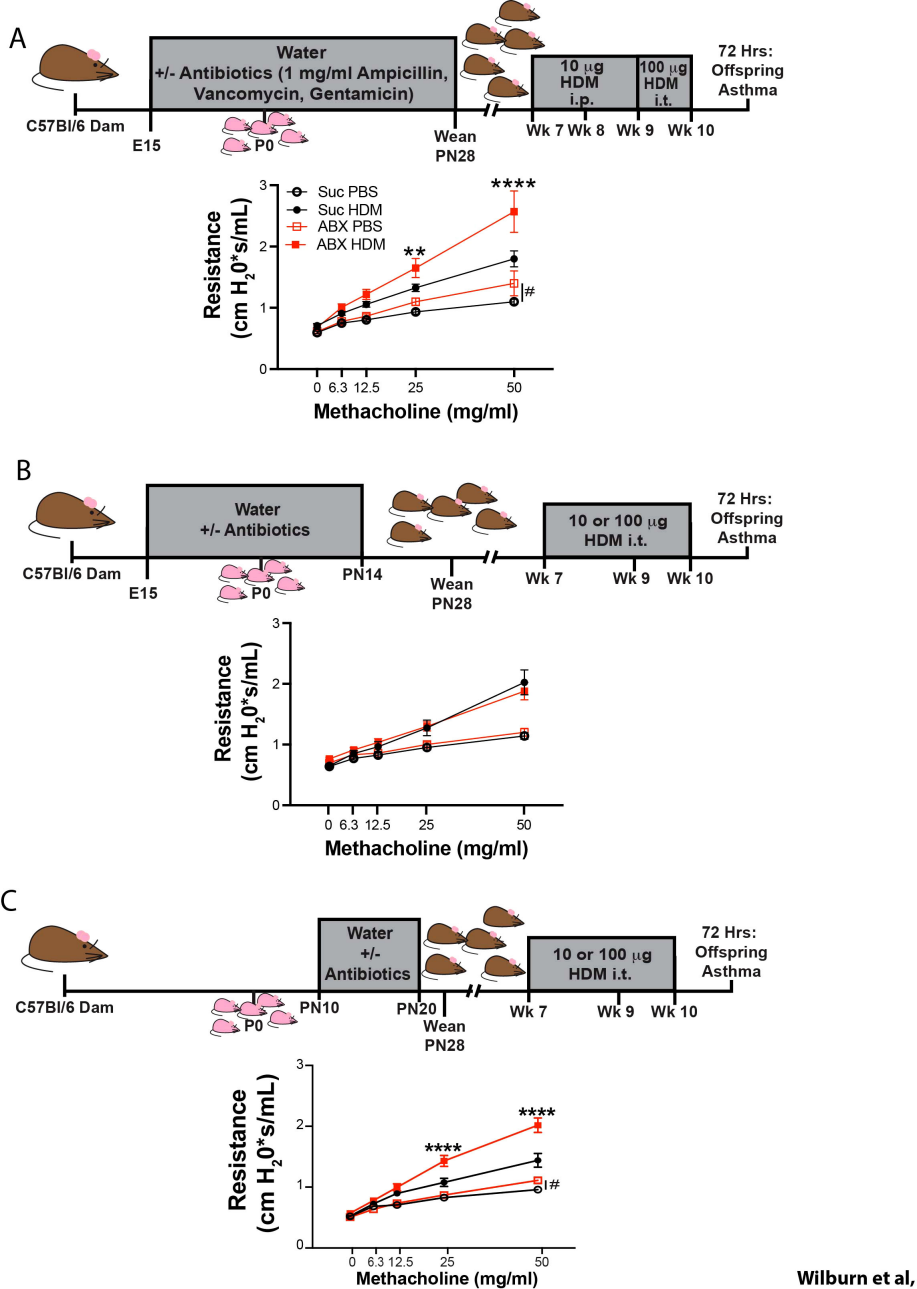
Dysbiosis during discrete developmental windows worsens allergen-induced AHR later in life. Nursing dams were given access to water supplemented with 0.5 mg/ml sucralose ± 1mg/ml each Ampicillin, Vancomycin, Gentamicin (red squares), or sucralose alone (black circles) from **(A)** embryonic day 15 (E15) to postnatal day 28 (PN28) **(B)** E15 to PN14, or **(C)** PN10 to PN20. In panel A, mice were sensitized i.p. with PBS or 10 µg of HDM lot 315580 (containing 0.6 EU LPS and 0.67 µg DerP1) at 7 and 8 weeks of life and challenged with i.t. PBS or 100 µg of HDM lot 315580 (containing 76 EU LPS and 6.7 µg DerP1) at 9 and 10 weeks of life **(A)**. In **(B, C)**, offspring were treated with i.t. PBS or HDM at 7, 9 or 10 weeks of life. In two experimental replicates, mice were treated with 100 µg of HDM from 343205 (containing of 7.68 EU of LPS and 6.17 µg of DerP1) and in one experimental replicate, mice were treated with 10 µg of HDM from lot 381017 (containing 0.098 EU of LPS and 5.73 µg of DerP1). 100 µg of HDM from lot 343205, and 10 µg of HDM from lot 381017 were found to induce equivalent levels of AHR, inflammatory cell recruitment, and induction of Th2 and Th17 immunity **(B, C)**. 72 hours after the final PBS/HDM treatment, we assessed methacholine-induced airway hyperreactivity (AHR) by FlexiVent. MEAN ± SEM shown. Normality of datasets was confirmed (Kolmogorov-Smirnov test). ** and **** indicate p<0.01, and p<0.0001 respectively between HDM-treated offspring of control, and ABX-exposed dams (Two-way ANOVA followed by Tukey’s multi-comparisons test between all 4 groups). <ns/> indicates p<0.05 between PBS-treated offspring of control, and ABX-exposed dams (Two-way ANOVA followed by Sidak’s multi-comparisons test between all only PBS-exposed groups). **(A)** n = 7–14 mice/group, in 3 independent experiments, **(B)** n = 11–18 mice/group, in 4 independent experiments, **(C)** n = 8–13 mice per group from 3 independent experiments.

For allergen (house dust mite) exposure, two separate models were used. In both, mice were anesthetized with isofluorane (2.5%) and given 40μl phosphate buffered saline (PBS) +/- house dust-mite extract (HDM; Greer). For animals in the E15-PN14 and PN10-PN20 models (see **Figure 1**), adult mice (>6 week old) were given either 100 µg of HDM from lot 343205 (768.71 EU LPS/mg of protein, 61.65 μg of DerP1/mg of protein, equivalent to 7.68 EU of LPS and 6.17 µg of DerP1), or 10 µg of HDM extract from lot 381017 (9.84 EU LPS/mg of protein, 57.28 μg of DerP1/mg of protein, equivalent to 0.098 EU of LPS and 5.73 µg of DerP1). HDM was given intratracheally (i.t) at weeks 7, 9 and 10 of life. Animals euthanized 72 hours after the final HDM exposure to assess the induction of airway inflammation. Importantly, 100 µg of HDM from lot 343205, and 10 µg of HDM from lot 381017 were found to induce equivalent levels of AHR, inflammatory cell recruitment, and induction of Th2 and Th17 immunity.

For offspring in the E15 to PN0 (see **Supplementary Figure 1**), and E15 to PN28 models (see **Figure 1**), adult offspring (>6 weeks of age) were treated with 10 µg of HDM (or PBS) intraperitoneally (i.p.) at week 7 and 8 of life, and mice were treated with 100 µg of HDM (or PBS) i.t. at weeks 9 and 10 of life. In this model, HDM from lot 315580 (700.6 EU LPS/mg of protein, 66.6 μg of DerP1/mg of protein). Thus, 10µg of HDM provided 7.6 EU LPS/0.67 µg DerP1 i.p., while 100 g of HDM provided 76 EU LPS/6.7 µg DerP1 i.t.

Importantly, ABX exposure regimens augmented AHR in both exclusively i.t. HDM-exposure models (**Figure 1A**), and in mixed i.p./i.t. HDM-exposure models (**Figure 1C**), suggesting that the differences in the ability of ABX exposures to augment HDM-induced AHR were related to timing of the ABX exposure, and not the model of HDM-exposure. Further, the ability of ABX-exposure to augment HDM-induced AHR was not related to the lot of HDM used, as several lots were used throughout the study. Where indicated, 8 µg of IL-5 depleting antibodies (clone TRFK5), or Isotype controls (IgG1, clone HRPN, both from BioXCell), were administered i.p. in 20 µl PBS on postnatal days 9, 11 and 13.

All offspring of control and ABX-exposed dams were housed separately to avoid potential normalization of microbiomes between the two groups. Within each group, mice were randomly assigned to PBS and HDM-treatment groups such that PBS- and HDM-treated offspring were cohoused.

### Assessment of lung mechanics

*In vivo* lung function measurements were performed using the FlexiVent system (Emka Technologies). Mice were anaesthetized with 150–175 mg/kg Ketamine and 20–25 mg/kg Xylazine, and body temperature was maintained at 37 °C. To avoid spontaneous inhalation, 1 mg/kg pancuronium bromide was injected i.p. The trachea was exposed, and a small incision was made between the tracheal rings to insert a beveled 18 G cannula (adult mice) or 19 G (juvenile mice). Cannula and trachea were kept airtight by surgical suture. Mice were ventilated at 150 breaths per minute and a positive end-expiratory pressure of 3 cm H_2_O. The Forced Oscillation Technique (FOT) was used to assess total resistance of the respiratory system (R_RS_), Newtonian resistance (resistance attributable to large airways; R_N_), and tissue damping (resistance attributable to small airways; G). All parameters were assessed following aerosolization of PBS, or increasing concentrations of methacholine (6.25, 12.5, 25 and 50 mg/mL). Where indicated, PV loops were measured with a modified script. This script completed one deep inflation, followed by one PV loop, followed by two deep inflations. The maneuver slowly inflates the lungs in steps, from Positive-End Expiratory Pressure (PEEP; 3 cmH_2_O) to 30 cmH_2_O, and then deflate them in a similar way to generate a PV loop. Animals were then subject to methacholine inhalation as described above.

### Histological assessment of lung development

Lungs were perfused with ice cold PBS, and inflation fixed with 4% paraformaldehyde in PBS at a pressure of 25 cm H_2_O. Lung tissue was excised from the chest cavity and stored in 4% paraformaldehyde overnight at 4°C. Tissue was then washed in PBS, dehydrated through a series of washes in ethanol and embedded in paraffin blocks. To sample the lung tissue at multiple planes, a total of ten 5 µm sections were captured per lung with 10 sections discarded in between each captured section. The paraffin melted, the tissues were rehydrated, stained with hematoxylin and eosin, and cover slipped. One random 20 X field from the distal lung parenchyma from each slide was captured on an EVOS7000 microscope, resulting in a total n = 10 20X fields per lung. Mean linear intercept (MLI) measurements were analyzed using a macro developed by R.M. Tuder for MetaMorph (49).

### NMR-based metabolomics

SCFA quantifications were performed at Translational Metabolomics Facility (RRID: SCR_022636). The lyophilized stooled samples were prepared according to the previously published protocol (50). Briefly, samples were homogenized in cold PBS with 2.8 mm ceramic beads (VWR) and the supernatants were filtered using pre-washed 3 kDa spin filters (NANOSEP 3K, Pall Life Sciences). The NMR sample was prepared with 400 µL of fecal filtrate with NMR buffer containing internal standard, TMSP. The NMR experiments were conducted on a Bruker Avance II 600 MHz spectrometer with PPO Prodigy probe. SCFA assignment and quantifications were performed using Chenomx^®^ NMR Suite profiling software (Chenomx Inc. version 8.4) based on the TMSP (0.33 mM). The SCFA concentrations were normalized to the original dried fecal sample weights (µmole/g) prior to the statistical analysis.

### Gut microbiome characterization

Fecal samples were collected longitudinally from a cohort of offspring derived from littermate mothers on postnatal (PN) day 23, and at 7, and 10 weeks of age. Fecal samples were obtained by scruffing mice. DNA extraction was performed from 0.25g of a stool sample with Power Fecal DNA Isolation Kit^®^ by MO BIO^®^ per kit instructions. DNA concentration was measured using Qubit^®^. Amplified library generation was performed with Nextera XT^®^ adapters (Illumina, Inc), and sequencing was performed on an Illumina NovaSeq 6000 machine to obtain 150bp DNA paired end reads to a depth of approximately 20 million reads per sample. Kraken2 was used for taxonomic classification from metagenomic sequences to species level. After assigning reads to taxa, read counts were normalized using the function rrarefy from the vegan R package and the percentage of reads assigned at to each Family was determined.

### Statistical analysis

All data are expressed as mean ± SEM. Normality of datasets was tested (Kolmogorov-Smirnov test; GraphPad Prism 10). Once normality was established, dose response AHR data between 4 groups (HDM-induced AHR) were compared using two-way ANOVA followed by a Tukey’s multiple comparisons *post-hoc* test. Dose response AHR data, and cellular recruitment time courses between 2 groups (baseline lung mechanics in offspring of control, and ABX-exposed dams) were compared using two-way ANOVA followed by a Sidak’s multiple comparison *post-hoc* test. Single measurement data compared between 2 groups (mean linear intercept, inspiratory capacity) was compared using unpaired Student’s t test. Where indicated, a Mann-Whitney test was used to datasets that were determined not to follow a normal distribution.

## Results

### Indirect exposure to antibiotics during critical neonatal windows augments allergen-induced airway hyperresponsiveness later in life

Although multiple studies have demonstrated that antibiotic exposure leads to increased development of allergic asthma, we wished to examine the consequences of neonatal antibiotic exposure on adult allergen-induced AHR. To this end, we developed a model wherein offspring are indirectly exposed to antibiotics (ABX) due to maternal antibiotic exposure. The three ABX used, Ampicillin, Vancomycin and Gentamicin are safe for use in lactating mothers (51), and thus mimic a situation where maternal treatment allows indirect exposure to ABX in neonates. Dams were given water supplemented with ABX + sucralose (to counteract the bitterness of ABX) or with sucralose alone from embryonic day 15 (E15), to postnatal day 28 (PN28) (see schematic – **Figure 1A**). After weaning, adult offspring of control and ABX-exposed dams were treated with i.t. PBS or HDM at 7, 9 and 10 weeks of age. 72 hours after the final HDM treatment, airway function was assessed by FlexiVent. As expected, HDM-treated offspring displayed higher methacholine-induced lung resistance than PBS-exposed offspring (compare solid symbols to open symbols - **Figure 1A**). Importantly, HDM-induced AHR was elevated in offspring of ABX-exposed dams compared to control mice (compare solid red symbols, to solid black symbols - **Figure 1A**). Interestingly, PBS-treated offspring of ABX-exposed dams also displayed significantly elevated AHR compared to control mice (compare open red symbols to open black symbols – **Figure 1A**), suggesting that maternal ABX exposure alters both HDM-induced AHR and basal lung resistance in adulthood.

To better understand the developmental “window” during which maternal ABX exposure can influence HDM-induced AHR in adult offspring, we varied the timing of maternal ABX exposure. As changes in the microbiome prior to, or around birth have been suggested to influence the development of asthma (6–11), we examined the ability of exclusively pre-natal ABX exposure to alter asthma outcomes. In contrast to effects of more prolonged ABX exposure, prenatal maternal ABX exposure (E15-PN0) was not associated with worse HDM-induced AHR in adulthood, or changes in baseline lung function (**Supplementary Figure 1**). We then assessed the impact of combined pre- and early post-natal ABX exposure by extending the window of ABX application to between E15 and PN14 (**Figure 1B**). The magnitude of AHR in control and offspring of ABX-exposed dams was again comparable, indicating that even this more extensive window of ABX exposure was not sufficient to alter development of allergen-induced allergic inflammation later in life and suggesting that the “window of susceptibility” is within the late post-natal period. Strikingly, limiting maternal ABX exposure to the PN10-PN20 period replicated the impact of extended ABX administration (E15-PN28), enhancing basal lung resistance, and HDM-induced AHR in adulthood in offspring of ABX-exposed dams (**Figure 1C**). Both male and female offspring exhibited augmented allergen-induced AHR following ABX exposure between E15-PN28, and PN10-PN20 (**Supplementary Figure 2**).

**Figure 2:**
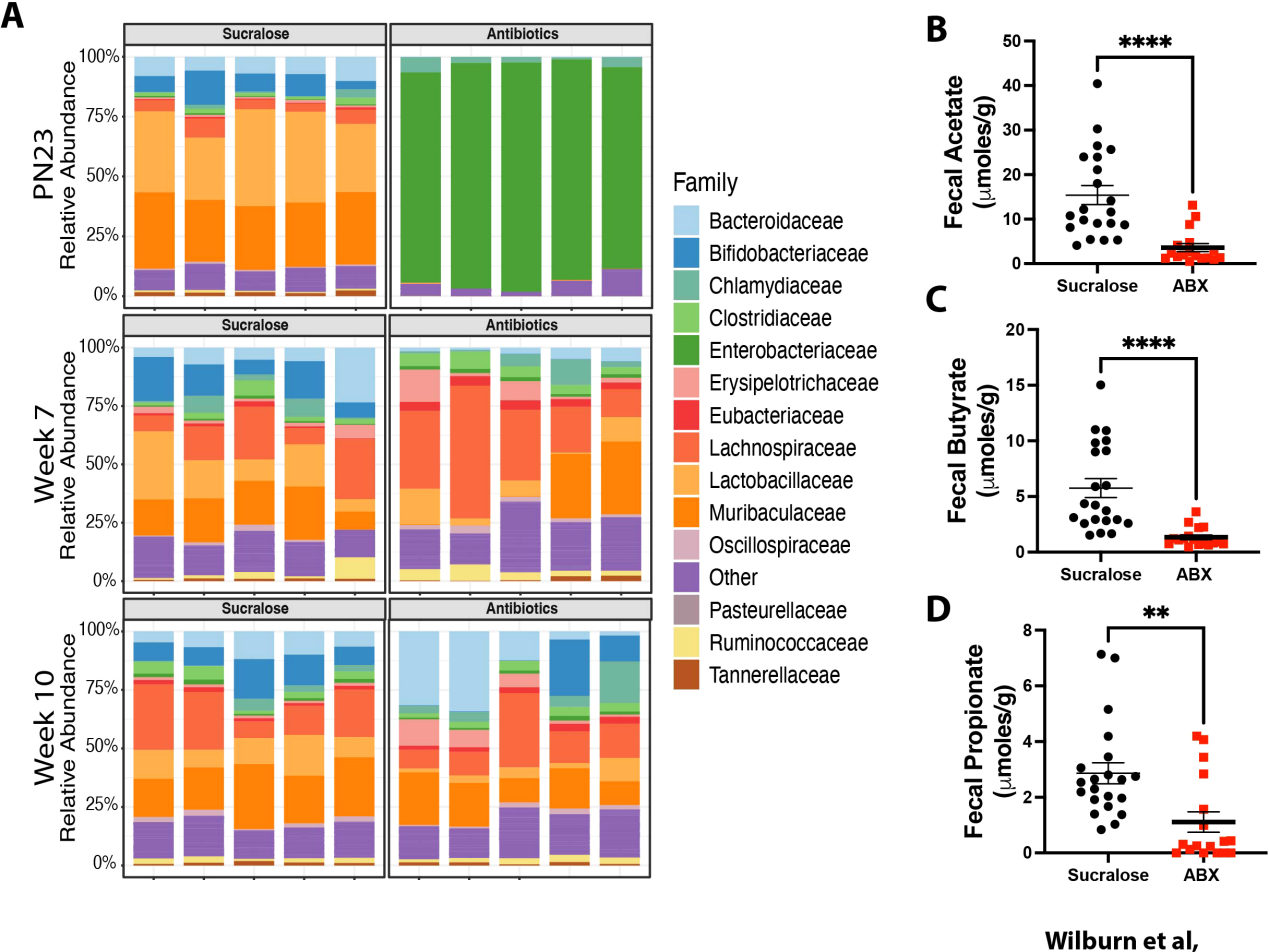
Maternal ABX exposure induces dysbiosis in offspring and is associated with reduced SCFA production. Nursing dams were given access to water supplemented with 0.5 mg/ml sucralose ± 1mg/ml each Ampicillin, Vancomycin, Gentamicin (red squares), or sucralose alone (black circles) from PN10 to PN20. Fecal samples were collected longitudinally from offspring at the indicated times and subjected to metagenomic analysis to identification of bacterial Families. n = 5 mice/group at each time point **(A)**. Fecal pellets were collected at PN28, and levels of **(B)** Acetate, **(C)** Butyrate, and **(D)** Propionate were measured by NMR. Mean ± SEM shown. As a Kolmogorov-Smirnov test demonstrated these data did not follow a normal distribution, a Mann-Whitney test was used. ** and **** indicate p<0.01 and p<0.0001 between Sucralose and ABX-exposed animals. n = 17–21 mice/group from 3 experiments.

Analysis of the gut microbiome composition in offspring of ABX-exposed dams confirmed induction of dysbiosis at PN23, as markedly reduced abundance of *Bacteroidaceae* and *Bifidobacteriaceae* and expansion of *Entereobacteriaceae* were observed (**Figure 2A**). The profound dysbiosis observed at PN23 was also associated with differential presence of key Short Chain Fatty Acids (SCFA) in the feces, as reduced levels of Acetate (**Figure 2B**), Butyrate (**Figure 2C**), and Propionate (**Figure 2D**) were observed at weaning (PN28). Importantly, dysbiosis was resolving at the time of allergen exposure (week 7), and nearly indistinguishable at time of sacrifice (week 10) (**Figure 2A**). Although male offspring of dams exposed to ABX between E15 and P28 had significantly reduced weight at 10 weeks of age (**Supplementary Figure 3**), similar decreases were not seen in female offspring of dams exposure to ABX between E15 and P28, or male and female offspring in any other ABX-exposure regimen. Thus, changes in baseline and allergen-induced AHR are unlikely to be due to differences in weight. Collectively, these data demonstrate that transient early life dysbiosis during a window between PN10 and PN20 is associated with augmented allergen-induced AHR later in life. These results demonstrate that transient, short-lived dysbiosis in neonates may drive the development of more severe allergen-induced AHR in adulthood.

### Changes in susceptibility to allergen-induced AHR are associated with durable alterations in baseline lung mechanics

**Figure 3:**
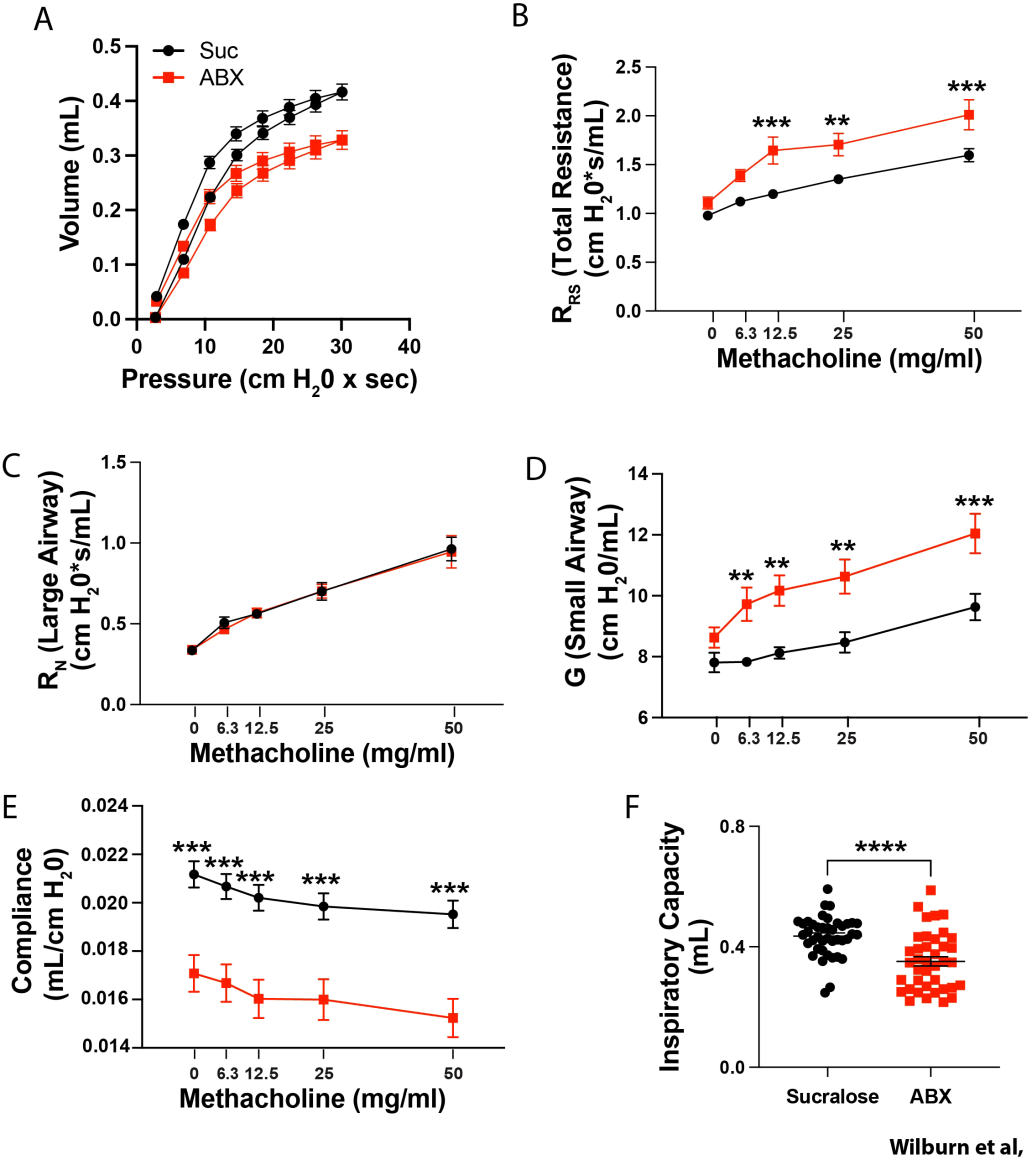
Maternal ABX exposure induces substantial changes in baseline lung mechanics in mice at weaning. Nursing dams were given access to water supplemented with 0.5 mg/ml sucralose ± 1mg/ml each Ampicillin, Vancomycin, Gentamicin (red squares), or sucralose alone (black circles) from PN10 to PN20. **(A)** Lung PV loops were assessed at PN28. After measuring PV-Loops, methacholine-induced changes in **(B)** the total resistance of the respiratory system (R_RS_), **(C)** Newtonian resistance (large airway resistance; R_N_), **(D)** Tissue damping, (small airway resistance; G), and **(E)** dynamic compliance (Crs) were assessed. **(F)** Inspiratory capacity (each dot represents an individual mouse) was also measured. Mean ± SEM shown. Normality of datasets was confirmed (Kolmogorov-Smirnov test). **, ***, and **** indicate p<0.01, p<0.001, and p<0.0001 between offspring of control, and ABX-exposed dams. **(B–E)** two-way ANOVA followed by Sidak’s multi-comparisons test, or **(F)** unpaired Student’s t test between offspring of control, and ABX-exposed dams. **(A)** n = 25–29 mice from 5 independent experiments, or **(B–F)** n = 27–38 mice from 7 independent experiments.

As evidence of altered lung mechanics has been reported in children prior to the development of asthma (45–48), we assessed whether changes in baseline lung mechanics preceded the development of more robust allergen-induced AHR. To this end, we more completely assessed lung mechanics at time of weaning (PN28) in offspring of control, and ABX-exposed dams. Assessment of PV loops from offspring of control ABX-exposed dams displayed a marked downward shift in the curve (**Figure 3A**) suggesting substantial changes in lung mechanics. Consistent with changes seen at 10 weeks of life, PN28 offspring of ABX-exposed dams displayed significantly elevated AHR compared to control animals (**Figure 3B**). Use of the FlexiVent “Forced Oscillation Technique (FOT)” further allows us to determine if changes are a result of small or large airway dysfunction (52, 53). Although large airway resistance (Newtonian resistance; R_N_) was comparable in offspring of control and ABX-exposed dams (**Figure 3C**), offspring of ABX-exposed dams displayed significantly elevated tissue damping (G), a measure of small airway resistance (**Figure 3D**). Offspring of ABX-exposed dams also had reduced dynamic compliance (Crs; **Figure 3E**) and inspiratory capacity (IC; **Figure 3F**). Interestingly, downward shifted PV loops, decreased compliance, and reduced respiratory capacity are changes observed in some fibrotic lung diseases (54, 55). Interestingly, baseline airway mechanics were not altered in offspring of dams exposed to either Ampicillin, Gentamicin or Vancomycin alone (**Supplementary Figure 4**) suggesting that changes in baseline lung function were not due to toxicity of any of the antibiotics tested. Collectively these data suggest that extensive changes in lung mechanics precede the development of more robust allergen-induced AHR.

**Figure 4:**
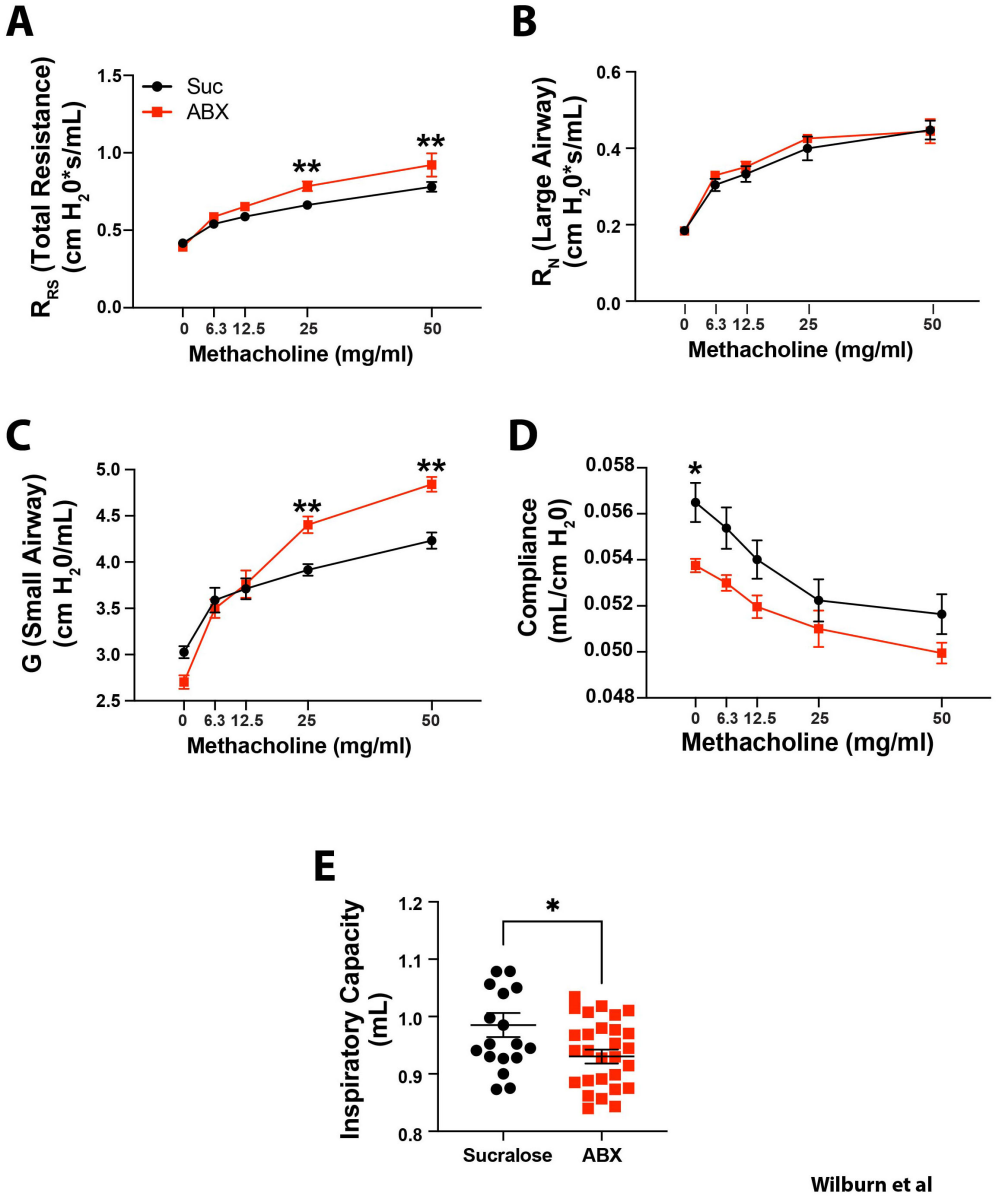
Maternal ABX exposure-induced changes in lung mechanics last until at least 14 months of age. Nursing dams were given access to water supplemented with 0.5 mg/ml sucralose ± 1mg/ml each Ampicillin, Vancomycin, Gentamicin (red squares), or sucralose alone (black circles) from PN10 to PN20. Offspring were aged until 14 months of age, and methacholine-induced changes in **(A)** the total resistance of the respiratory system (R_RS_), **(B)** Newtonian resistance (large airway resistance; R_N_), **(C)** Tissue damping, (small airway resistance; G), and **(D)** dynamic compliance (Crs) were assessed. **(E)** Inspiratory capacity (each dot represents an individual mouse) was also assessed prior to methacholine exposure. Mean ± SEM shown. Normality of datasets was confirmed (Kolmogorov-Smirnov test). ** indicates p<0.001 between offspring of control, and ABX-exposed dams. **(A–D)** Two-way ANOVA followed by Sidak’s multi-comparisons test, or **(E)** unpaired Student’s t test between offspring of control, and ABX-exposed dams. n = 9–12 mice from 4 independent experiments. * indicates p < 0.05, and ** indicates p < 0.01.

To examine the durability of altered lung mechanics, similar measurements were made in 14 month-old mice. Consistent with the observations that neonatal lungs display inherently more resistance than adult lungs (56), total (R_rs_), large (R_N_) and small airway resistance (G) were all lower in adult animals than neonates (**Figure 4**). Remarkably, when offspring of control and ABX-exposed dams were compared, a similar pattern of increased R_rs_ (**Figure 4A**), comparable R_N_ (**Figure 4B**), increased G (**Figure 4C**), decreased Crs (**Figure 4D**), and lower Inspiratory Capacity (**Figure 4E**) was observed in 14 month-old offspring of ABX-exposed dams. These results indicate that maternal ABX exposure between PN10 and PN20 has a profound, and long-lasting effect on lung mechanics.

### Maternal ABX exposure alters the trajectory of alveolar development, and is associated with augmented recruitment of eosinophils to the neonatal lung

**Figure 5:**
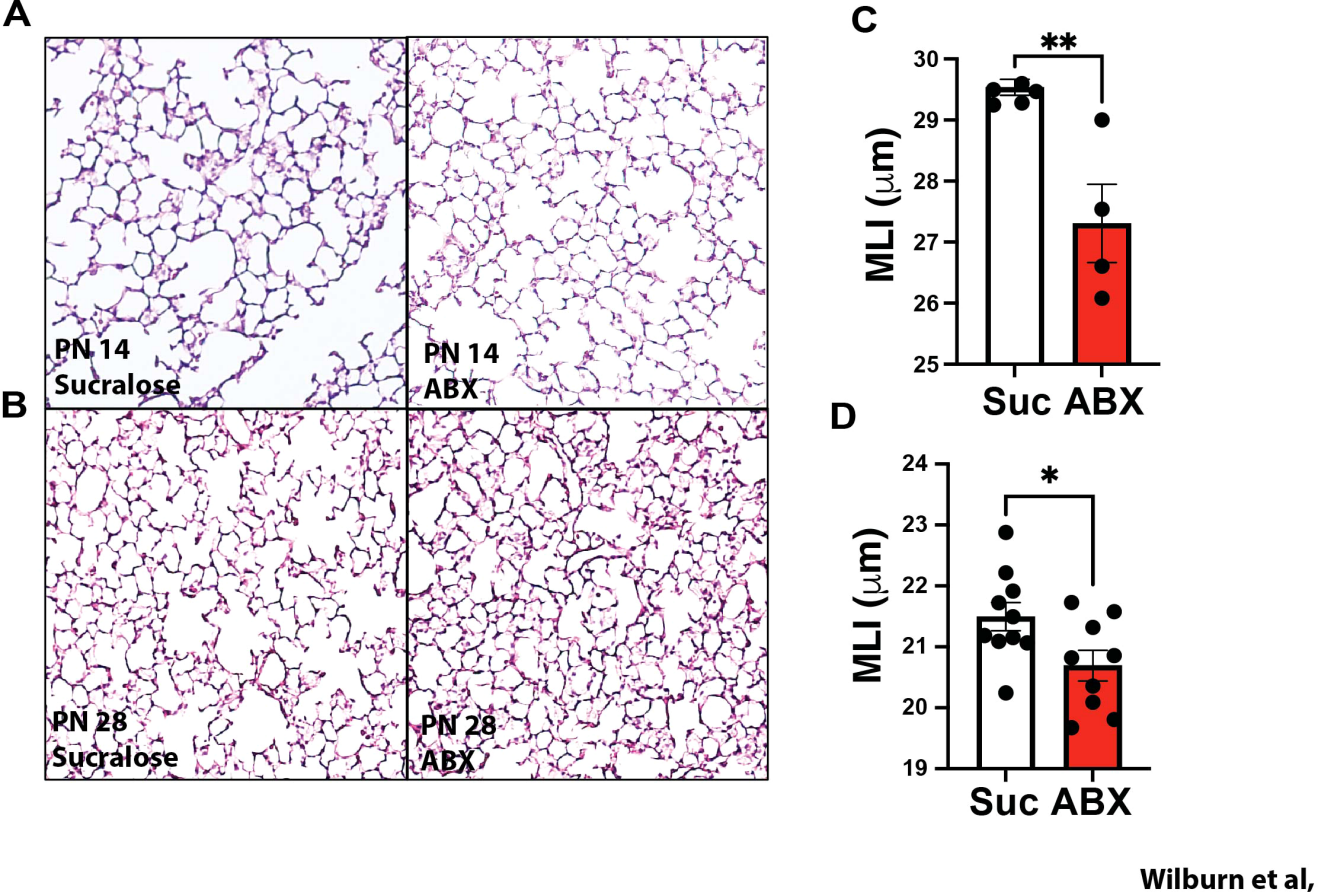
Maternal ABX exposure during critical developmental window impacts the rate of alveolar development. Nursing dams were given access to water supplemented with 0.5 mg/ml sucralose ± 1mg/ml each Ampicillin, Vancomycin, Gentamicin (red squares), or sucralose alone (black circles) from PN10 to PN20. Lungs were inflation fixed at **(A)** PN14 or **(B)** PN 28 and alveolar development was assessed by histology. Representative histological images taken from offspring of control (left panel), or ABX-exposed dams (right panels). Mean linear intercept (MLI), a measure of alveolar size, was assessed in sections from multiple offspring of control (open bars) or ABX-exposed dams (red bars) at **(C)** PN14 and **(D)** PN28. Mean ± SEM shown. Normality of datasets was confirmed (Kolmogorov-Smirnov test). * and ** indicate p<0.05 and p<0.01 between offspring of control and ABX-exposed dams. Each dot indicates data from a single mouse. PN14: Suc n=5, ABX n=4; PN28: suc n=10, ABX n=9.

Given the marked impact of maternal ABX exposure on lung mechanics observed at weaning, and in aged mice, we next compared the structural development of the lung in offspring of control and ABX-exposed dams. As changes in lung mechanics appeared “small airway-focused” (increased G, but normal R_N_) and our window of maternal ABX exposure overlaps with an increased rate of alveolarization observed between PN10 to 18 (12), we performed a focused histological assessment of alveolar structure at PN14 and PN28. While PN14 represents a period of dynamic and robust alveolarization, this process is more advanced by PN28. Consistent with this, alveoli appeared smaller and more numerous at PN28 compared to PN14 (**Figures 5A, B**). Interestingly, offspring of ABX-exposed dams appeared to have smaller, more numerous alveoli at both time points, an observation confirmed by a significantly decreased Mean Linear Intercept (MLI) at both PN14 (**Figure 5C**), and PN28 (**Figure 5D**). The reduced MLI observed in offspring of ABX-exposed dams suggests that maternal ABX exposure was potentially accelerating the normal trajectory of alveolarization.

**Figure 6:**
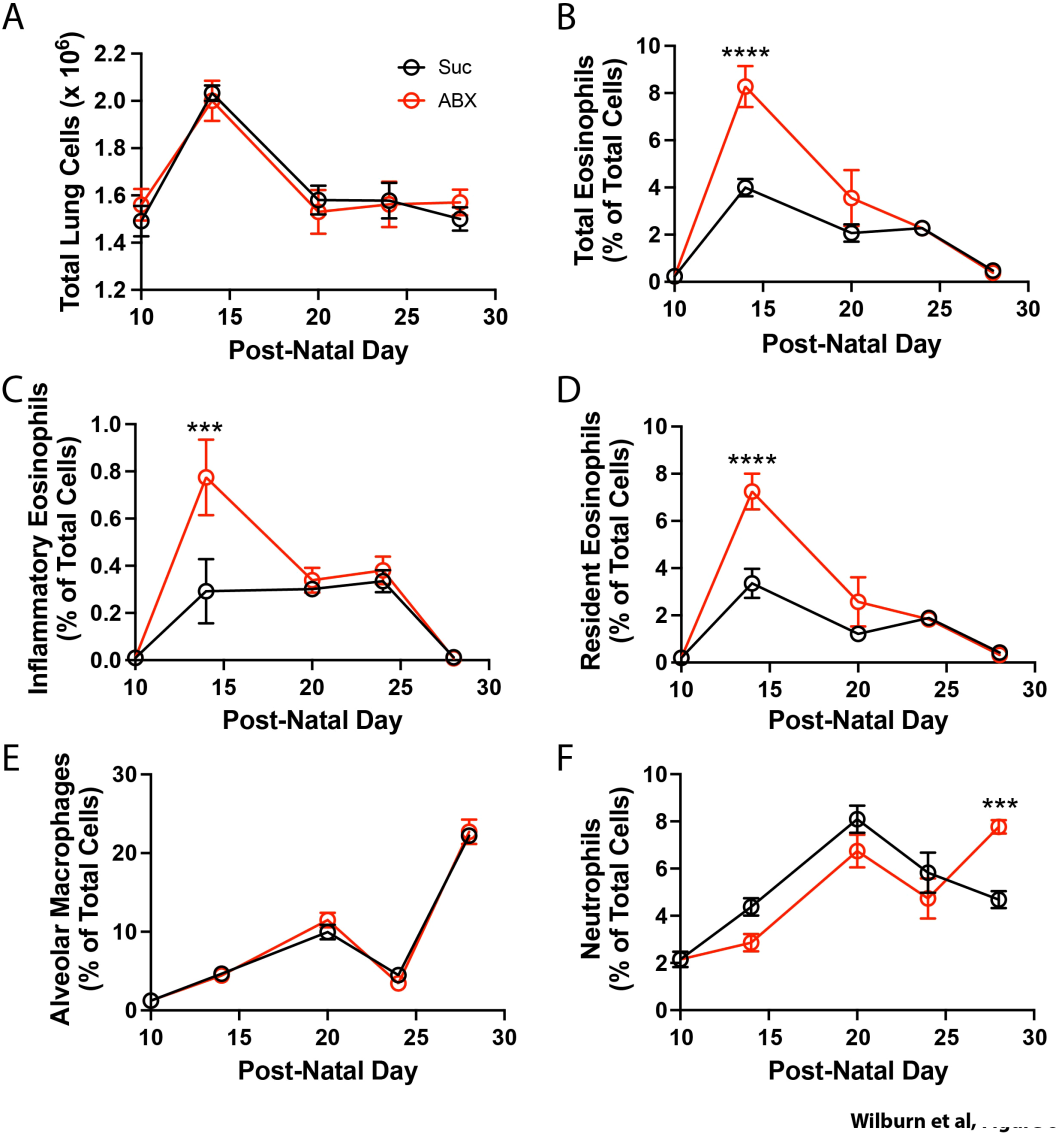
Maternal ABX exposure increases recruitment of eosinophils into the neonatal lung. Nursing dams were given access to water supplemented with 0.5 mg/ml sucralose ± 1 mg/ml each Ampicillin, Vancomycin, Gentamicin (red symbols), or sucralose alone (black symbols) from PN10 to PN20. Lungs were removed prior to the induction of dysbiosis (PN10), or at various times during the period of dysbiosis, and single cell suspensions were generated. Total lung cells were enumerated **(A)**. Percentage of total eosinophils **(B)**, inflammatory eosinophils **(C)**, resident eosinophils **(D)**, alveolar macrophages **(E)** and neutrophils **(F)** in the lungs were assessed by flow cytometry. MEAN ± SEM shown. Normality of datasets was confirmed (Kolmogorov-Smirnov test). ***, and **** indicate p<0.001, and p<0.0001 between offspring of control, and ABX-exposed dams. n = 14 mice (P10); 3–10 mice (P14); 6–9 mice (P20); n = 6–8 mice (P24); n = 6–9 mice (P28).

It has been hypothesized that homeostatic lung remodeling between PN10 to PN14 is supported by recruitment of Th2-associated cells (ILC2s, eosinophils, macrophages) to the neonatal lungs (15–19). To determine if altered lung development/function in offspring of ABX-exposed dams was associated with different patterns of inflammatory cell recruitment, we assessed the presence of macrophages and granulocytes in the lungs of offspring of control and ABX-exposed dams between PN10 and PN28 (see **Supplementary Figure 5** for gating strategy). A spike in total lung cellularity was observed at PN14, and this returned to PN10-like levels by PN20 (**Figure 6A**). Importantly, numbers of cells recovered from the lungs remained comparable in offspring of control, and ABX-exposed dams (**Figure 6A**). We noted a rise in the frequency of eosinophils in the lung at PN14, followed by a steady decline in eosinophil numbers until PN28, at which time they had returned to levels seen at PN10 (**Figure 6B**). Importantly, offspring of ABX-exposed dams demonstrated more robust recruitment of eosinophils to the lung at PN14 (**Figure 6B**), after which eosinophil levels declined following the same kinetics as observed in offspring of control dams. As both inflammatory eosinophils (iEos) and resident eosinophils (rEos) have been described (57, 58), we also determined if maternal ABX exposure altered the type of eosinophils recruited to the neonatal lung (see **Supplementary Figure 5** for gating strategy). In offspring of both control, and ABX-exposed dams, the “wave” consisted primarily of rEos (~90%), with the remaining ~10% expressing markers consistent with iEos (**Figures 5C, D**). Alveolar macrophages increased in frequency steadily throughout this period (**Figure 5E**) with comparable numbers observed in offspring of control dams. Numbers of neutrophils peaked at PN20 in offspring of both control and ABX-exposed dams, but there were significantly more neutrophils in the lungs of offspring of ABX-exposed dams at PN28 (**Figure 5F**). Back gating on identified populations suggested that populations represented distinct cell types: neutrophils were of uniformly low complexity/small size, alveolar macrophages demonstrated a much larger size, and a wide range of complexity, and eosinophils (both iEos, and rEos) were highly complex and slightly larger than neutrophils (**Supplementary Figure 6**). These parameters are consistent with expectations for these cells types. Finally, adoption of an alternative gating strategy which identified eosinophils as SiglecF^bright^CD11c^low^ (iEos CD101^neg^; rEos CD101^+^) and alveolar macrophages as SiglecF^bright^CD11c^bright^ was employed (See **Supplementary Figures 7A, B**). This gating strategy demonstrated consistent results – with an increase of total eosinophils, iEos, and rEos, observed in offspring of ABX-exposed dams at PN14, with no impact on the frequency of alveolar macrophages in the lungs at this time (**Supplementary Figure 7C**). Collectively, these data demonstrate that maternal exposure to ABX alters the recruitment of eosinophils to the developing lung.

### Mice lacking eosinophils display altered basal lung mechanics at weaning

**Figure 7:**
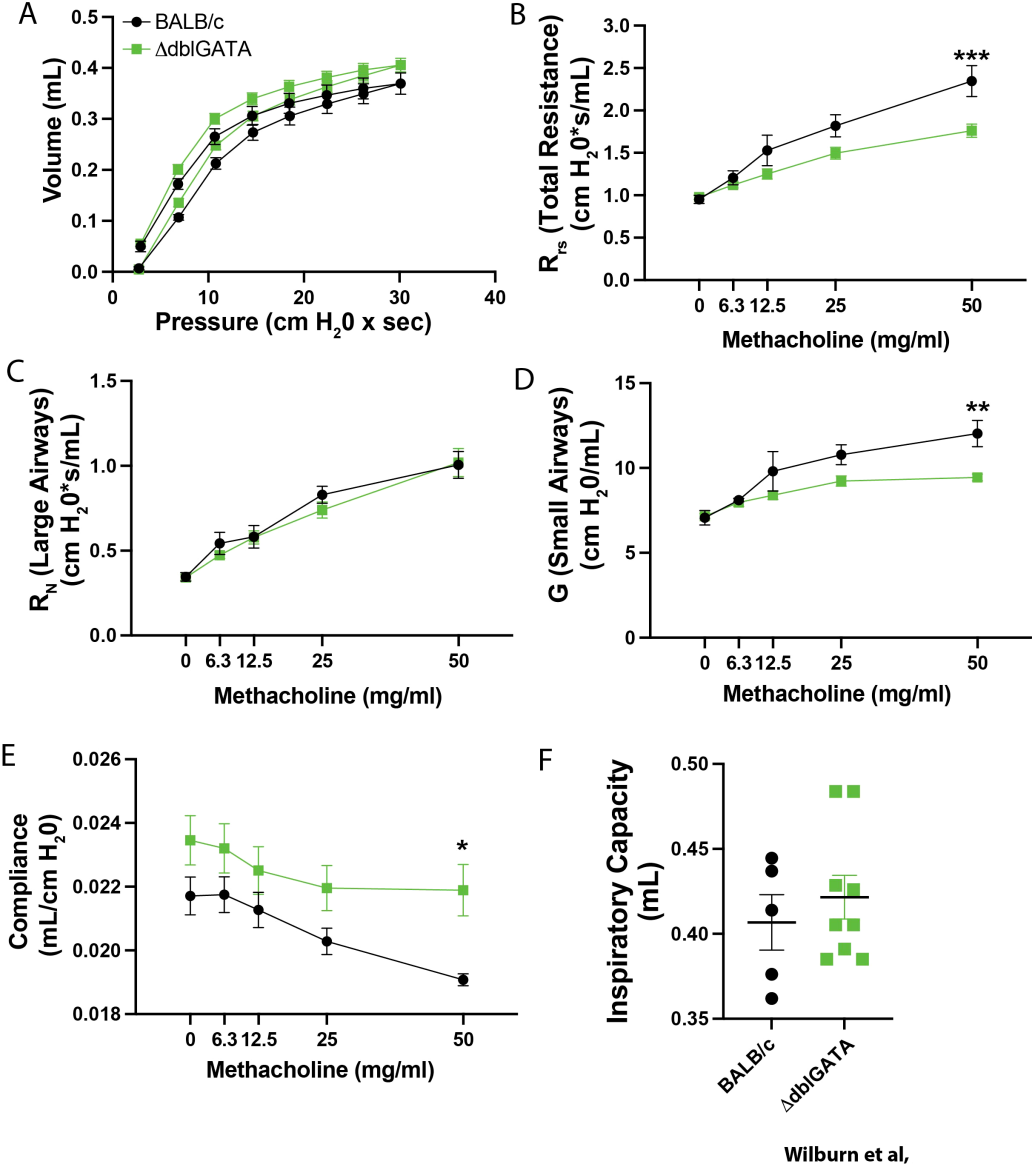
Eosinophil deficient mice display altered lung mechanics. Lung mechanics were assessed in PN28 BALB/c (black symbols) and ΔdblGATA mice (green symbols) by FlexiVent. **(A)** Lung PV loops were assessed. After measuring PV-Loops, methacholine-induced changes in **(B)** the total resistance of the respiratory system (R_RS_), **(C)** Newtonian resistance (large airway resistance; R_N_), **(D)** Tissue damping (small airway resistance; G), and **(E)** dynamic compliance (Crs) were also assessed. **(F)** Inspiratory capacity (each dot represents an individual mouse) was also measured. Mean ± SEM shown. Normality of datasets was confirmed (Kolmogorov-Smirnov test). *, **, and *** indicate p<0.05, p<0.01, and p<0.001 between BALB/c and ΔdblGATA. n = 5–9 mice per group.

To explore the role of eosinophils in regulating basal lung function in neonates, we assessed lung mechanics in WT BALB/c, and eosinophil deficient (ΔdblGATA) BALB/c mice at PN28. ΔdblGATA neonates demonstrated an upward shifted PV loop (**Figure 7A**) associated with decreased Rrs (**Figure 7B**), unaltered RN (**Figure 7C**), decreased G (**Figure 7D**), and reduced Crs (**Figure 7E**), and a trend towards increased IC (**Figure 7F**). Importantly, this phenotype is opposite to that observed in offspring of ABX-exposed dams. As an alternative approach, αIL-5 (8 µg/dose), or isotype control was given to WT mice on PN9, PN11, and PN13 to acutely deplete eosinophils, and lung mechanics, and MLI was assessed at PN37. αIL-5 almost completely abrogated the recruitment of eosinophils (both inflammatory and regulatory) in the lung at PN14, without impacting that of neutrophils or alveolar macrophages (**Supplementary Figure 8**) demonstrating that the increase in homeostatic eosinophil numbers in the neonatal lung is IL-5-dependent. Consistent with observations in ΔdblGATA mice, αIL-5 treated mice tended to have upward shifted PV loops, reduced R_rs_ with unaltered R_N_, and reduced G, as well as increased Crs, and elevated Inspiratory Capacity (**Supplementary Figure 8**).

An alternative possibility, that ABX-exposure induced changes in offspring growth might contribute to observed changes in lung function was also considered. To examine this, neonates weight in various groups was assessed (**Supplementary Figure 9**). Although maternal ABX exposure to the cocktail of ABX between PN10 and PN20 was associated with significantly reduced weight (and increased baseline Rrs) in both male, and female offspring at PN28, offspring of dams exposed to a single ABX tended to also display reduced weight. As maternal exposure to a single ABX did not change baseline Rrs (**Supplementary Figure 4**) ABX-exposure induced changes in lung function are not likely simply due to lower offspring weight. Similarly, weight was not different in αIL-5 treated mice (which displayed decreased baseline lung Rrs), and ΔdblGATA mice (which displayed reduced baseline Rrs) displayed reduced weight. As such, there seems to be no consistent relationship between offspring weight, and baseline lung mechanics. Taken together, these data suggest that eosinophils play an underappreciated role in lung development and provide additional insights into mechanisms through which early life exposures may influence disease development later in life.

## Discussion

The hypothesis that dysbiosis influences asthma development is based on human data spanning 25 years (6–11). This work is supported by key animals studies. Importantly, our study is unique in several ways. Because germ free mice (utilized in a number of studies (24–28)) lack all microbiome-derived signals, it is unclear whether they represent a state of dysbiosis, or broader immune dysfunction. In occasions where antibiotics were utilized this frequently occurred after weaning (29–32, 35, 37, 38, 40), thus missing key perinatal windows identified in human studies (8–11, 59–70). When antibiotics were given during the perinatal period, this often was also associated with additional post-natal exposure (33, 41), allergen sensitization challenge occurred during the period of dysbiosis (34), or ABX were applied as a method of depleting endogenous flora prior to transfer of fecal contents from other mice (36). In situations where exclusively perinatal ABX were applied and allergen sensitization occurred in mice after normalization of the microbiome, the direct impact of microbial exposures, or the impact of dysbiosis on basal lung mechanics was not determined (20, 21). Interestingly, it has been reported that germ free mice have increased alveolar size (71) suggesting a direct impact of microbial colonization on homeostatic lung development. Additionally, compared to germ free mice reconstituted with microbiota from control mice, those reconstituted with microbiota from azithromycin- or amoxicillin-exposed mice demonstrated a significant increase in baseline Rrs (39). However, our study is the first, to our knowledge, to demonstrate a potential link between maternal ABX-exposure, eosinophil recruitment, and alterations in lung development and mechanics.

We observe that offspring of dams treated with ABX within a specific neonatal window between PN10 and PN20 display life-long changes in basal lung mechanics (downward shifted PV loops, increased total and small airway resistance, and decreased dynamic compliance and inspiratory capacity) and are prone to the development of more severe AHR after allergen exposure. Although, many of the changes observed in baseline lung function are consistent with changes seen in fibrotic lung diseases (downward shifted PV loops, reduced compliance, decreased inspiratory capacity (54, 55)), we observed no evidence of increased collagen deposition that would be a hallmark of fibrosis in the neonatal lungs at PN28 (**Supplementary Figure 10**). Unfortunately, we were unable to compare collagen deposition in 14 month-old offspring of control and ABX-exposed dams; as fibrotic changes often require longer term models to develop, these animals may have been more likely to demonstrate differences. Nonetheless, the observed changes in lung development and small airway function are consistent with modifications to the normal trajectory of alveolar development, a process going on in the lung during between PN14 to 28 (12, 13). Our observation of smaller, more numerous alveoli in offspring of ABX-exposed dams, suggests an acceleration of alveolar development. Although we have not directly linked early life airway dysfunction to the development of asthma in later life, these observations are consistent with reports demonstrating that altered patterns of lung development can precede the development of asthma (42–48). Moreover, altering alveolar epithelial cell function can enhance the development of allergen-induced AHR (72, 73), suggesting that alterations in the process of alveolarization may have underappreciated effects on development of allergen-induced responses later in life. The mechanisms responsible for altered baseline lung mechanics, and the contributions that these changes have on susceptibility to the development of asthma later in life, remain an area of active investigation.

In both mice, and humans, extensive remodeling and maturation occurs in the post-natal period (13, 14). The accumulation of Th2-associated immune cells (ILC2s, eosinophils, alveolar macrophages) has been described in the neonatal mice immediately after birth, and again at around post-natal day 14 (15–19). The functional role of these cells, at these times, has not been fully explored, but they may contribute to lung development in multiple ways. Eosinophils in particular release cytokines and growth factors such as IL-4, IL-13, and TGF-β, which can modulate epithelial cell differentiation, fibroblast activity, and extracellular matrix organization (74, 75), suggesting a potential role in tissue homeostasis. Eosinophils contribute to homeostatic development of mammary glands in postnatal (76), and post-pregnancy (77) periods, as well as in the development of small intestinal villi (78), and adipose tissue (79). Although excessive eosinophil activity in the fetal and neonatal lung has been linked to susceptibility to the development of chronic lung diseases (45, 80–82) the contributions of eosinophils to basal lung mechanics and homeostatic alveolar morphogenesis has not been interrogated before. This is surprising, given that mice completely lacking eosinophils were developed more than 20 years ago (83, 84). Indeed, these strains have been studied primarily to dissect the role of eosinophil in pathogenesis of allergic asthma; their role in baseline lung mechanics, or development have not been a primary area of interest. However, a careful review of the literature does demonstrate “non-overlapping” pulmonary function curves in allergen-naïve control mice and mice lacking eosinophils, or major eosinophil products (85–89), although this is, admittedly, not universally observed (83). Similarly, subtle changes in the mesenchymal expression of proteins associated with normal extra cellular matrix expression/deposition have also been reported in mice lacking eosinophils (90). Our observations of altered kinetics of lung development, lung mechanics and asthma susceptibility in mice with altered numbers of eosinophils (either enhanced (offspring of ABX-exposed dams) or reduced (ΔdblGATA mice, αIL-5 treated mice)) supports the hypothesis that disruption of eosinophil recruitment during periods of homeostatic remodeling interferes with the trajectory of tissue development, and may predispose to long-term functional deficits and heightened susceptibility to asthma later in life.

Our observations of increased recruitment of eosinophils into the lungs of offspring of ABX-exposed dams suggest that microbial signals can influence eosinophil recruitment. However, the systemic signals through which maternal ABX exposure alters eosinophil recruitment remain unclear. It has been reported that prenatal maternal ABX exposure during a limited pre-natal window (E10 to E14) augmented eosinophil recruitment between PN7 to PN21, and could worsen papain-induced asthma (91). These changes were found to be driven by ABX-induced epigenetic-driven alterations in ILC2 function which increased their capacity to produce Th2 cytokines (91). These results are consistent with other reports suggesting that the microbiome can regulate lung ILC2 activity (92–94). In contrast, in our model, ABX exposure between PN10 and PN20 is not associated with more robust ILC2 activity following allergen exposure (23). This may be related to our choice of allergen (HDM is less ILC2 dependent than papain (95, 96)), but we hypothesize that the timing of ABX exposure is also important. It is likely that ILC2 precursors are already well established by PN10, and therefore less susceptible to epigenetic-mediated regulation of function due to maternal exposure at E10 to E14 (91). Regarding regulation of eosinophil activity, short chain fatty acids (SCFA) derived from fermentation of dietary fiber by ABX-sensitive bacteria (Acetate, Butyrate, Propionate) can directly limit eosinophil migration, gene expression, and induce eosinophil apoptosis (97), and have anti-allergic effects (98, 99). Importantly, we see profound reductions in the levels of these SCFA in the feces of offspring of ABX-exposed dams (**Figures 2B-D**), suggesting altered availability of these mediators in our offspring. We speculate that reduced SCFA levels are facilitating increased eosinophil development, recruitment or activity in our models, which then drive more robust and accelerated alveolar development. Nonetheless, these observations argue that availability of microbiome-derived factors at different developmental windows can have unique effects on immune cell activity/recruitment, with varied effects on the trajectory of tissue and susceptibility to chronic lung diseases. This is an area of ongoing investigation in our lab.

Our work is the first to suggest that reduced eosinophil activation/recruitment during discrete developmental periods may lead to maladaptive trajectories of lung development. Importantly, observations related to lung mechanics and development from models of low eosinophil numbers are largely the opposite of those from models of hyperactive or excessive eosinophil recruitment, suggesting a consistent role for eosinophils as a cell type that is a driver of these homeostatic processes. Although there is no evidence of acute safety issues associated with the use of eosinophil targeting therapies (anti-IL-5, anti-IL-5R) at conception or in pregnancy (100), and that these therapies are generally well-tolerated in children, with safety profiles similar to those observed in adults (101, 102), alveolar development has been reported to extend out to 15 years of age in humans (14). Thus, if eosinophils do control homeostatic lung development that occurs between the ages of 6 and 15, anti-eosinophil targeting therapies may have subtle impacts on lung development that may alter susceptibility to the development of chronic diseases later in life. Clearly further studies are warranted to determine if, and when similar waves of eosinophils are recruited to the lungs (or other tissues) in humans, and whether they are similarly IL-5 dependent.

One limitation of this study is that our most intriguing association – that increased altered lung mechanics are observed only in animals that develop severe AHR following allergen exposure in adulthood – remains mechanistically untested. While broadly consistent with studies demonstrating increased asthma risk in those with altered trajectories of lung development or function (42–48), our observations would imply that dysregulated eosinophil recruitment in the neonatal period was the mechanism responsible for altered asthma phenotype later in life. It should be noted that this may provide a mechanistic explanation for why severe neonatal respiratory viral infections that drive eosinophil recruitment (i.e. RSV) (103–105) are associated with worse asthma outcomes later in life (103, 106, 107). Importantly, the sensitivity of neonatal eosinophil recruitment to αIL-5 affords us the opportunity to control the level of eosinophil recruitment into the developing lung with precision. Ongoing studies in the lab seek to reduce the levels of eosinophils observed in the offspring of ABX-exposed dams, bringing them back to levels observed in control dams, and then examining the magnitude of allergen-indued AHR that develops later in life.

Our findings collectively demonstrate that maternal ABX exposure during lactation can influence the normal trajectory of lung development and lung mechanics. The changes observed in both lung mechanics and structure in offspring of mothers exposed to ABX are centered in the alveolar region of the lung – consistent with the major homeostatic processes that are ongoing at the time of ABX exposure in our model. Alterations in the trajectory of lung development are further associated with increased susceptibility to the development of allergic asthma and appear to be driven by the increased recruitment of eosinophils, during these developmental windows. Thus, our work highlights novel roles of innate immune cells in lung development and uncovers underappreciated effects of maternal ABX exposure and eosinophils on developmental processes in the lung.

## Data Availability

The raw data supporting the conclusions of this article will be made available by the authors, without undue reservation.
