# Supplementary material for: Maternal antibiotic exposure-mediated alterations in basal, and allergen-induced lung function are associated with altered recruitment of eosinophils to the developing lung
Source: Front Immunol. 2025 Dec 18;16:1715675. doi: 10.3389/fimmu.2025.1715675 (PMC12756434; doi:10.3389/fimmu.2025.1715675)
Supplement: Supplementary file 1 [file DataSheet1.docx]

**Supplementary Materials and Methods:**

*Lung Collagen Measurements:*

Offspring of control and ABX-exposed dams were euthanized at PN28, lung harvested, and lysates generated. Total (soluble and insoluble) lung collagen was measured using a hydroxyproline kit, per manufacturer’s directions (Sigma).

**Supplementary Figure Legends:**

**Supplementary Figure 1. Maternal ABX exposure between E15 and PN0 does not influence allergen-induced AHR in offspring.** Nursing dams were given access to water supplemented with 0.5 mg/ml sucralose ± 1mg/ml each Ampicillin, Vancomycin, Gentamicin (red squares), or sucralose alone (black circles) from E15 to PN0. Mice were sensitized i.p. with PBS or 10 µg of HDM lot 315580 (containing 0.6 EU LPS and 0.67 µg DerP1) at 7 and 8 weeks of life and challenged with i.t. PBS or 100 µg of HDM lot 315580 (containing 76 EU LPS and 6.7 µg DerP1) at 9 and 10 weeks of life. 72 hours after the final PBS/HDM treatment, we assessed methacholine-induced airway hyperreactivity (AHR) by FlexiVent. MEAN ± SEM shown. n = 11 – 18 mice/group, in 4 independent experiments.

**Supplementary Figure 2. Maternal ABX exposure between PN10 and PN20 augments allergen-induced AHR in male and female offspring.** (A, B) Nursing dams were given access to water supplemented with 0.5 mg/ml sucralose ± 1mg/ml each Ampicillin, Vancomycin, Gentamicin (red squares), or sucralose alone (black circles) from E15 to PN28. Female (A) and male (B) mice were sensitized i.p. with PBS or 10 µg of HDM lot 315580 (containing 0.6 EU LPS and 0.67 µg DerP1) at 7 and 8 weeks of life and challenged with i.t. PBS or 100 µg of HDM lot 315580 (containing 76 EU LPS and 6.7 µg DerP1) at 9 and 10 weeks of life. (C, D) Nursing dams were given access to water supplemented with 0.5 mg/ml sucralose ± 1mg/ml each Ampicillin, Vancomycin, Gentamicin (red squares), or sucralose alone (black circles) from PN10 to PN20. Female (C) and male (D) offspring were treated with i.t. PBS or HDM at 7, 9 or 10 weeks of life. In two experimental replicates, mice were treated with 100 µg of HDM from 343205 (containing of 7.68 EU of LPS and 6.17 µg of DerP1) and in one experimental replicate, mice were treated with 10 µg of HDM from lot 381017 (containing 0.098 EU of LPS and 5.73 µg of DerP1). 72 hours after the final PBS/HDM treatment, we assessed methacholine-induced airway hyperreactivity (AHR) by FlexiVent.

**Supplementary Figure 3. Maternal ABX exposure does not consistently alter offspring weight in adulthood.** Nursing dams were given access to water supplemented with 0.5 mg/ml sucralose ± 1mg/ml each Ampicillin, Vancomycin, Gentamicin or sucralose alone (black circles from (A) embryonic day 15 (E15) to postnatal day 28 (PN28) (B) E15 to PN0, (C) E15 to PN14, or (D) PN10 to PN20. Weight of offspring was assessed in male and female offspring of control, and ABX-exposed dams prior to measurement of airway responses at week 10 of life. Normality of datasets was confirmed (Kolmogorov-Smirnov test). * indicates p<0.05 between offspring of control, and ABX-exposed dams (Student’s t test). Each dot represents a single mouse. n = 7 – 10 mice (A), n = 16 – 43 mice (B), n = 12 – 18 mice (C), n = 26 – 39 mice (D).

**Supplementary Figure 4. Maternal exposure between PN10 and PN2- to a single ABX does not alter baseline lung mechanics.** Nursing dams were given access to water supplemented with 0.5 mg/ml sucralose ± 1mg/ml Ampicillin (A), Gentamicin (B) or Vancomycin (C) between PN10 and PN20. AT PN28, mice were sacrificed for assessment of total resistance of the respiratory system (R_RS_), Newtonian resistance (large airway resistance; R_N_), and Tissue damping. N = 8 – 10 mice (A), n = 8 – 9 mice (B) or n = 7 – 10 mice (C).

**Supplementary Figure 5. Flow cytometry gating strategy.** Nursing dams were given access to water supplemented with 0.5 mg/ml sucralose ± 1mg/ml each Ampicillin, Vancomycin, Gentamicin or sucralose alone between PN10 to PN20, and lungs were removed at the indicated times. Single cell suspensions were generated and stained for flow cytometric analysis. (A) Representative flow plots show identification of singlets and cells. (B) Subsequent gating allowed the identification of Neutrophils (PMNs), Alveolar macrophages (Alv Mac), total eosinophils, as well as “inflammatory” and “resident” eosinophil populations. Representative flow plots from representative offspring of control (top panels), or ABX-exposed dams (bottom panel). (C) FMO plots for Siglec F and CD125 to support gating of PMN population. (D) FMO plots for CD101 and CD11c to support gating of Total Eosinphils and Alveolar Macrophage populations.

**Supplementary Figure 6. Back gating of cell populations identified by flow cytometry reveal distinct physical characteristics.** Flow populations identified as Neutrophils (A), Alveolar Macrophages (B) and Eosinophils (C) in Supplementary Figure 5 were back gated to examine complexity (SSC) and size (FSC).

**Supplementary Figure 7. An alternative gating strategy to identify Eosinophils and Alveolar Macrophages confirms offspring of ABX-exposed dams have an increased frequency of Eosinophils at PN14.** Nursing dams were given access to water supplemented with 0.5 mg/ml sucralose ± 1mg/ml each Ampicillin, Vancomycin, Gentamicin or sucralose alone between PN10 to PN20, and lungs were removed at the indicated times. Single cell suspensions were generated and stained for flow cytometric analysis. (A) An alternative gating strategy to identify Eosinophils and Alveolar Macrophages. This gating strategy was used to quantify the frequency of immune cells in the lungs of offspring of control (black symbols/lines) and ABX-exposed (red symbols/lines) dams. MEAN ± SEM shown. Normality of datasets was confirmed (Kolmogorov-Smirnov test). *, **, and *** indicate p<0.05, p<0.01, p<0,001 between offspring of control, and ABX-exposed dams. n = 14 mice (P10); 3 – 10 mice (P14); 6 – 9 mice (P20); n = 6 – 8 mice (P24); n = 6 – 9 mice (P28).

**Supplementary Figure 8. Anti-IL-5 administration selectively depletes eosinophils in the neonatal lung.** Mice were treated with 8 µg of IL-5 blocking mAb (clone TRFK5; red bars) or isotype control mAb (Rat IgG1, κ; black bars) on days PN9, PN11, and PN13. (A) Lungs were collected at PN14 and the frequency of granulocyte (Total eosinophils, inflammatory eosinophils, resident eosinophils and neutrophils) and alveolar macrophages were assessed by flow cytometry. At PN28, mice were euthanized to measure (B) PV Loops, (C) methacholine-induced changes in the total resistance of the respiratory system (R_RS_), (D) Newtonian resistance (large airway resistance; R_N_), (E) Tissue damping (small airway resistance; G) (F) dynamic compliance (Crs) and (G) inspiratory capacity (each dot represents an individual mouse). Mean ± SEM shown. Normality of datasets was confirmed (Kolmogorov-Smirnov test). * indicates p<0.05 between isotype- and αIL-5-treated mice. (A) n = 2 – 3 mice/group from 1 representative experiment of 2 completed. (B) n=7 mice/group from a single experiment. (C–G) 10 – 14 mice from 3 independent experiments.

**Supplementary Figure 9. Decreased weight of offspring of dams exposed to ABX between PN10 and PN20 is not associated with altered airway function.** Between PN10 and PN20 nursing dams were given access to water supplemented with 0.5 mg/ml sucralose ± 1mg/ml each Ampicillin, Vancomycin, Gentamicin (A), Ampicillin alone (B), Gentamicin alone (C) or Vancomycin alone (D) and weight of offspring was assessed at PN28. (D) Mice were treated with Isotype or αIL-5 on days PN9, PN11, and PN13, and weight of offspring was assessed at PN37. (E) weight of WT BALB/c and ΔdblGATA mice was assessed at PN28. MEAN ± SEM shown. Normality of datasets was confirmed (Kolmogorov-Smirnov test). * indicates p<0.05 between groups.

**Supplementary Figure 10. Comparable collagen levels in lungs of offspring of control, and ABX-exposed dams.** Nursing dams were given access to water supplemented with 0.5 mg/ml sucralose ± 1mg/ml each Ampicillin, Vancomycin, Gentamicin or sucralose alone between PN10 to PN20, and lungs were removed PN28. Levels of total collagen (soluble and insoluble) were assessed as described in Materials and Methods. Mean ± SEM shown. n = 15 – 16 mice/group from 2 experiments.

**Supplementary References:**

1. Jee JJ, Yang L, Shivakumar P, Xu PP, Mourya R, Thanekar U, Yu P, Zhu Y, Pan Y, Wang H, Duan X, Ye Y, Wang B, Jin Z, Liu Y, Cao Z, Watanabe-Chailland M, Romick-Rosendale LE, Wagner M, Fei L, Luo Z, Ollberding NJ, Tang ST, Bezerra JA. Maternal regulation of biliary disease in neonates via gut microbial metabolites. *Nat Commun* 2022; 13: 18.
