# Supplementary figures and images for: Maternal antibiotic exposure-mediated alterations in basal, and allergen-induced lung function are associated with altered recruitment of eosinophils to the developing lung
Source: Front Immunol. 2025 Dec 18;16:1715675. doi: 10.3389/fimmu.2025.1715675 (PMC12756434; doi:10.3389/fimmu.2025.1715675)

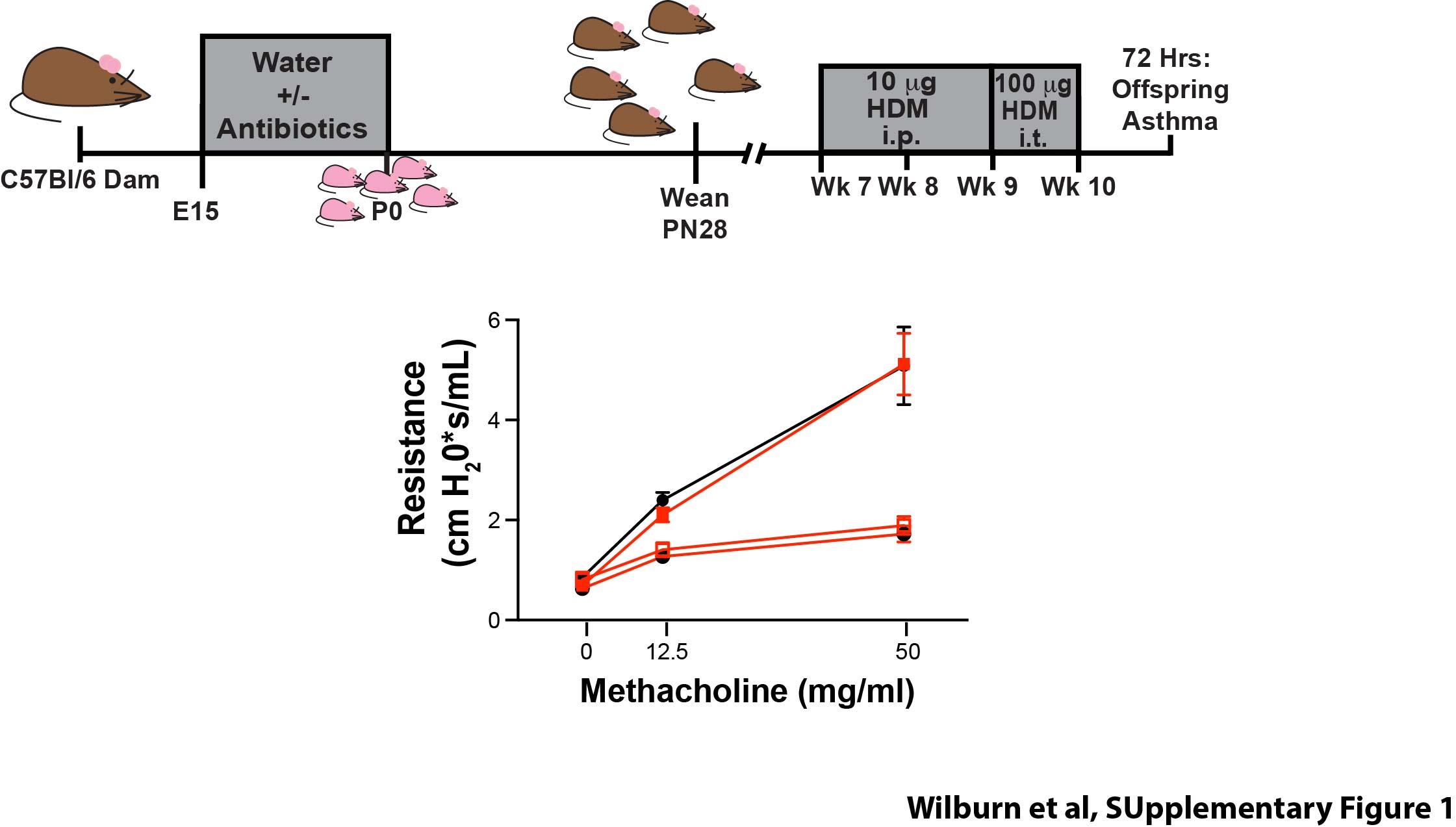

Supplement: Supplementary file 2 [file Image1.jpeg]

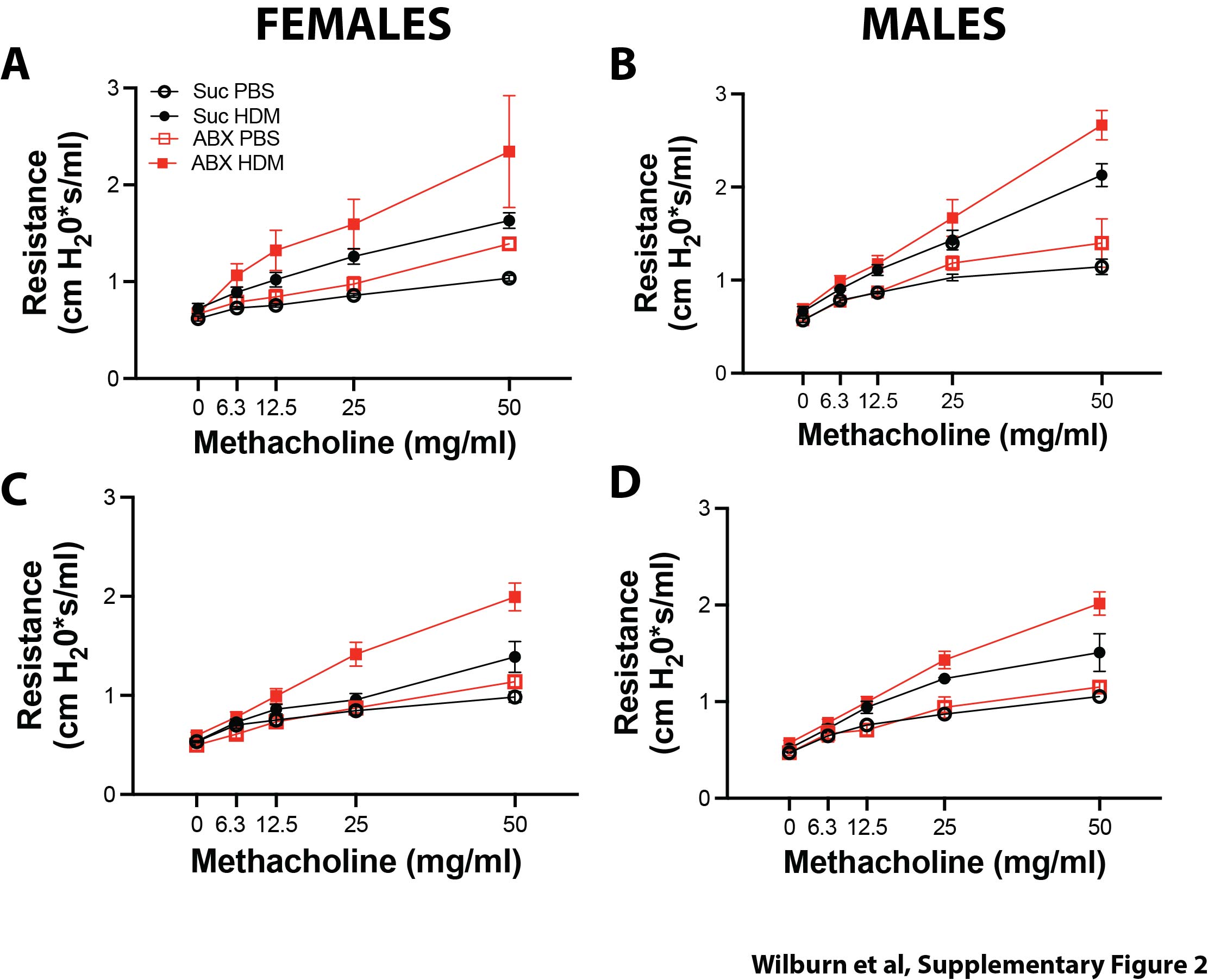

Supplement: Supplementary file 3 [file Image2.jpg]

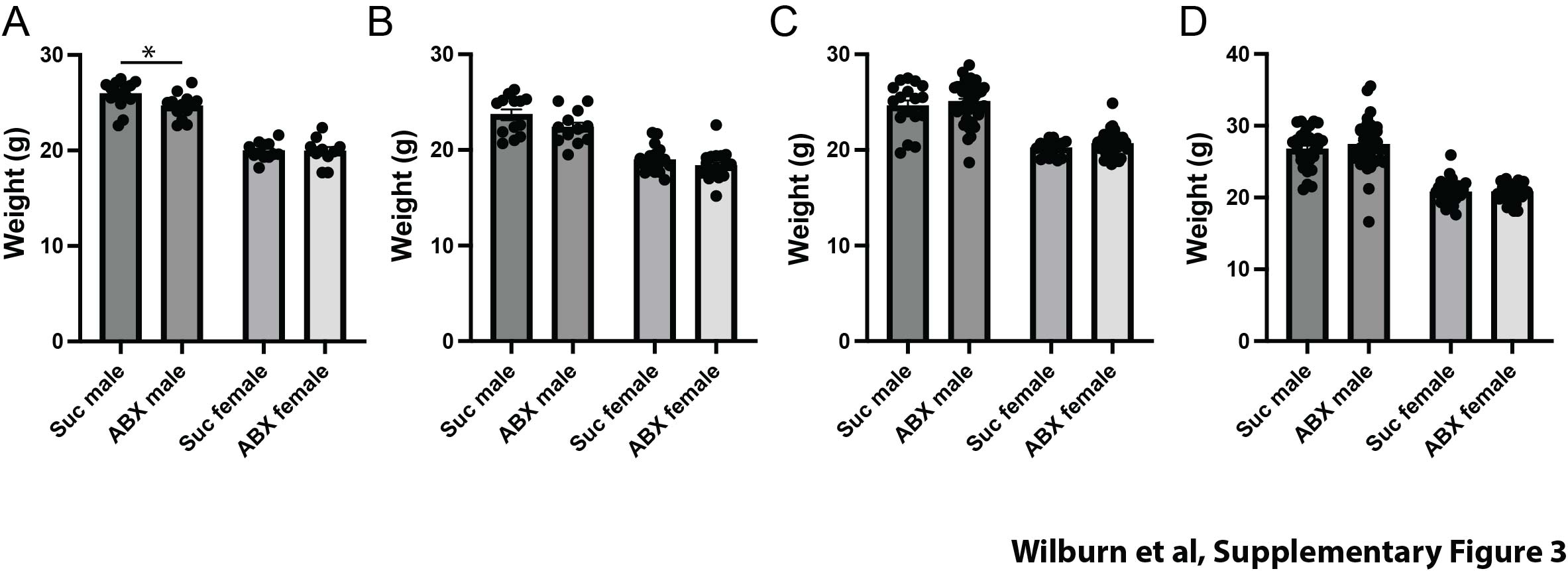

Supplement: Supplementary file 4 [file Image3.jpeg]

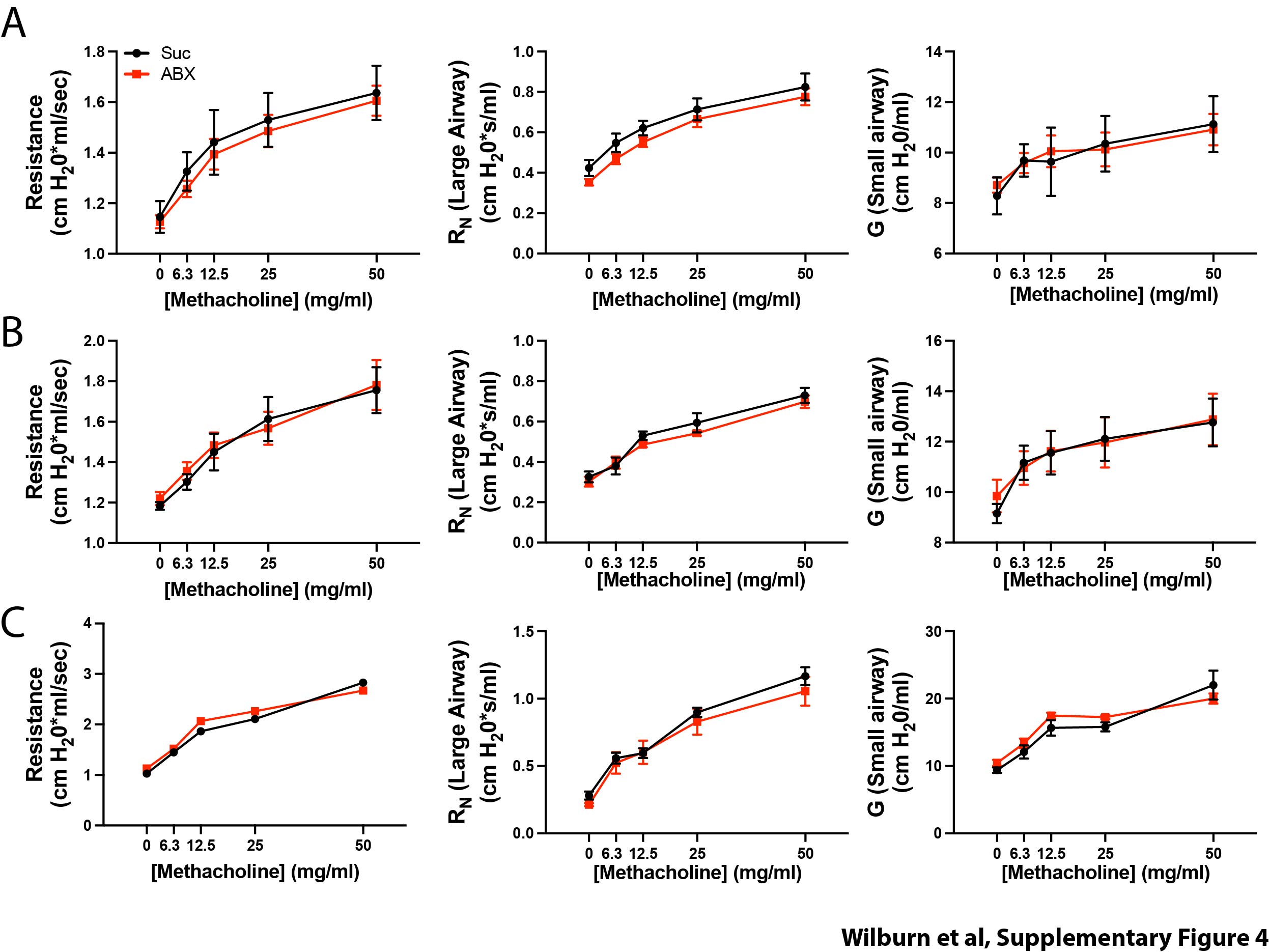

Supplement: Supplementary file 5 [file Image4.jpeg]

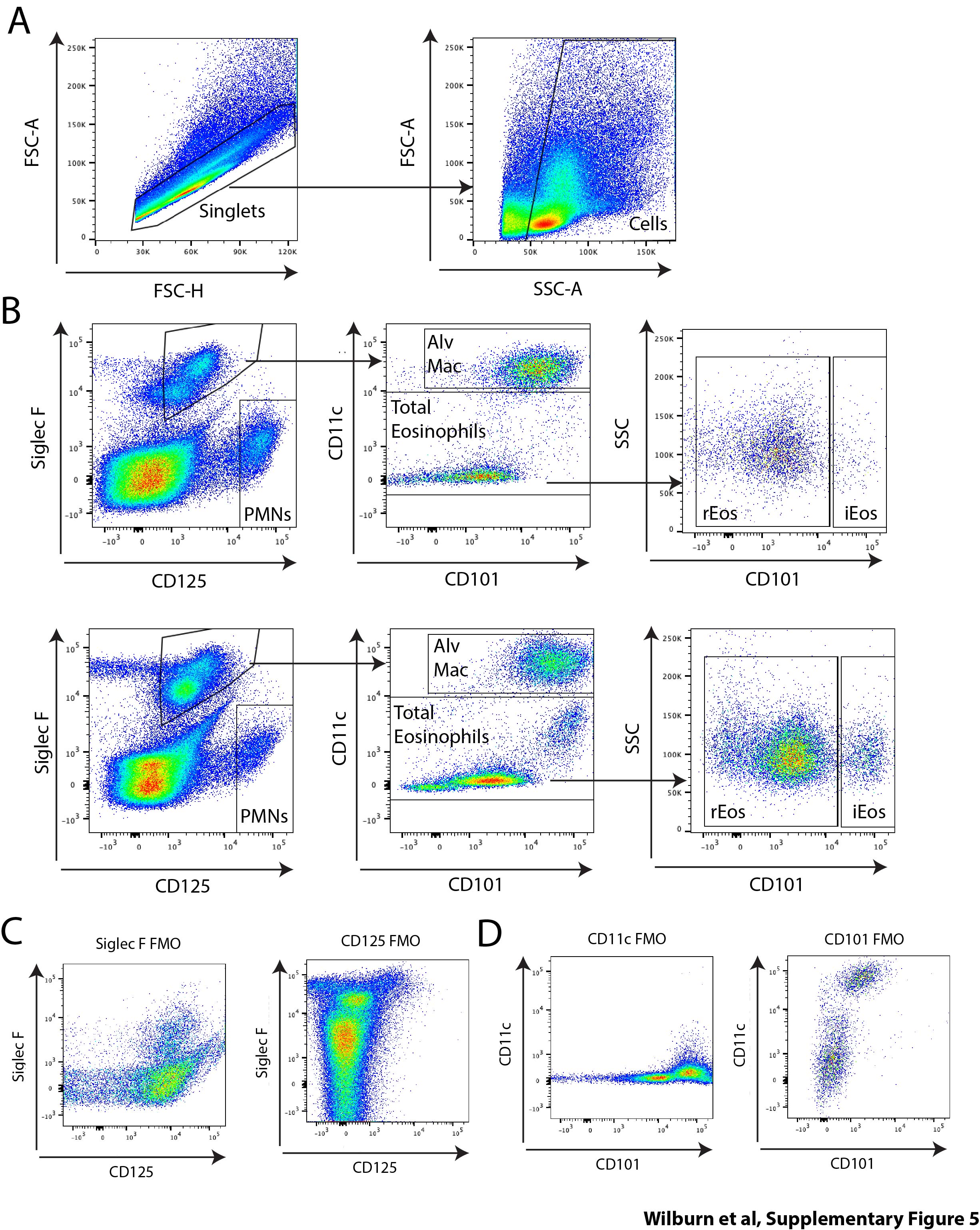

Supplement: Supplementary file 6 [file Image5.jpeg]

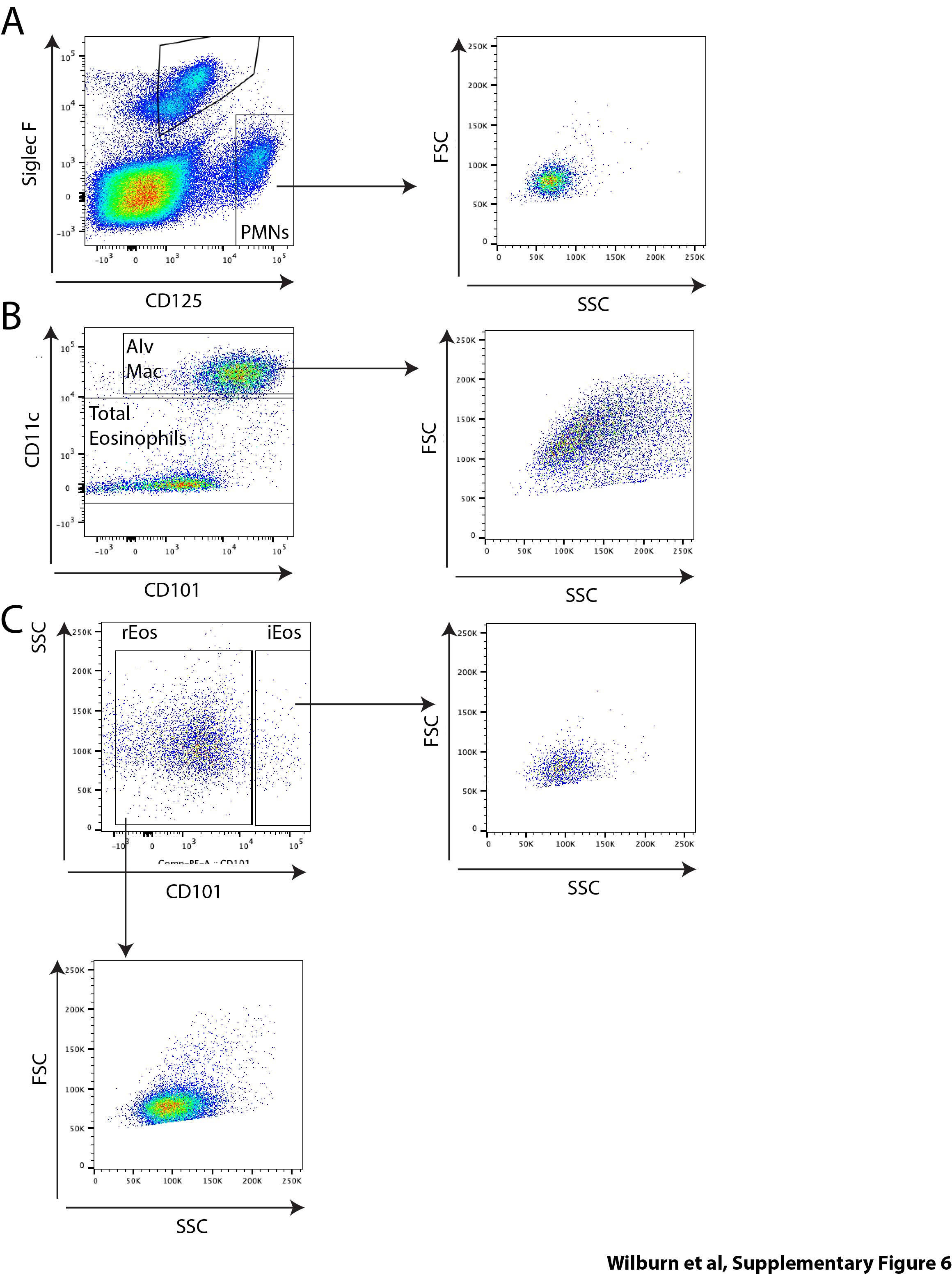

Supplement: Supplementary file 7 [file Image6.jpeg]

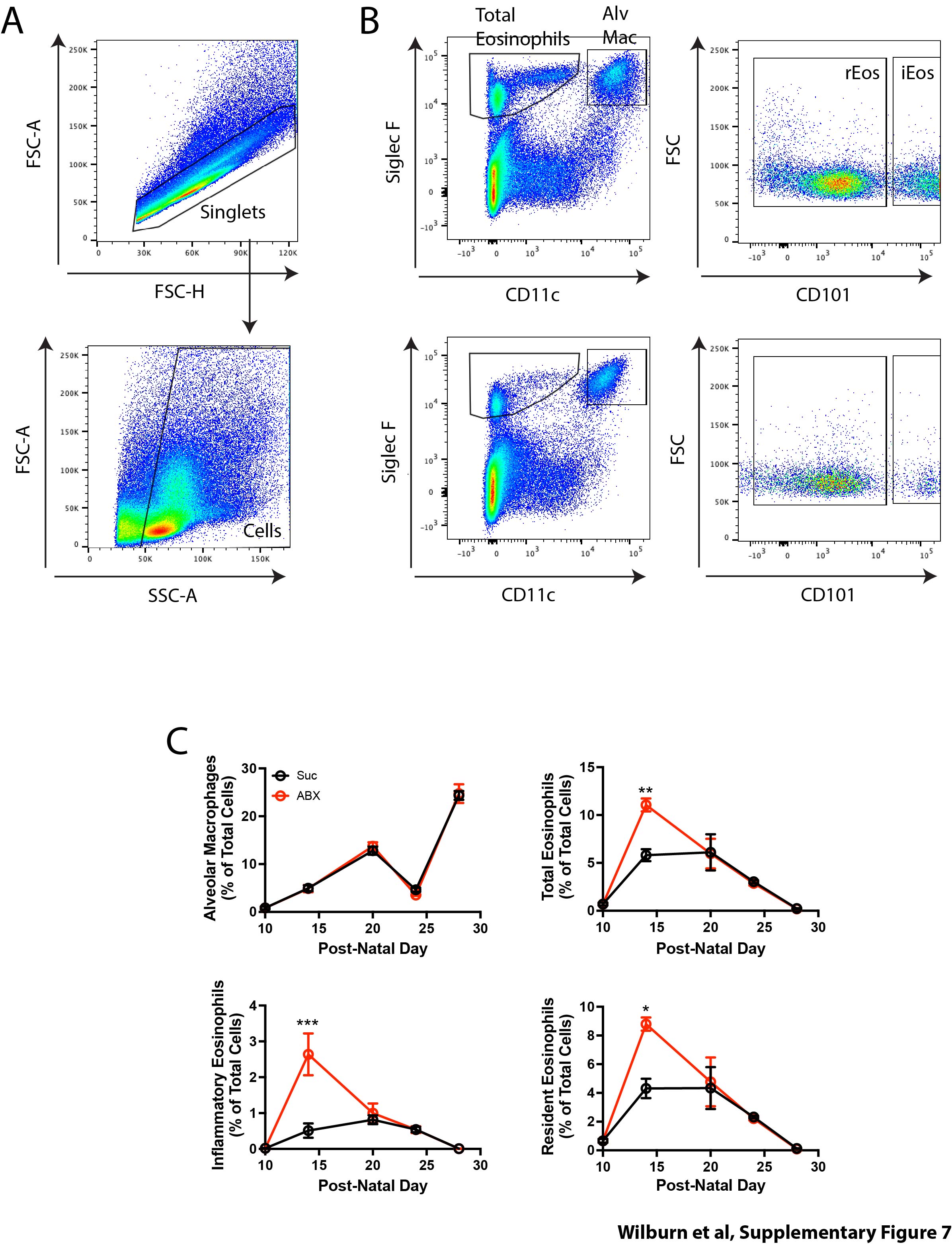

Supplement: Supplementary file 8 [file Image7.jpeg]

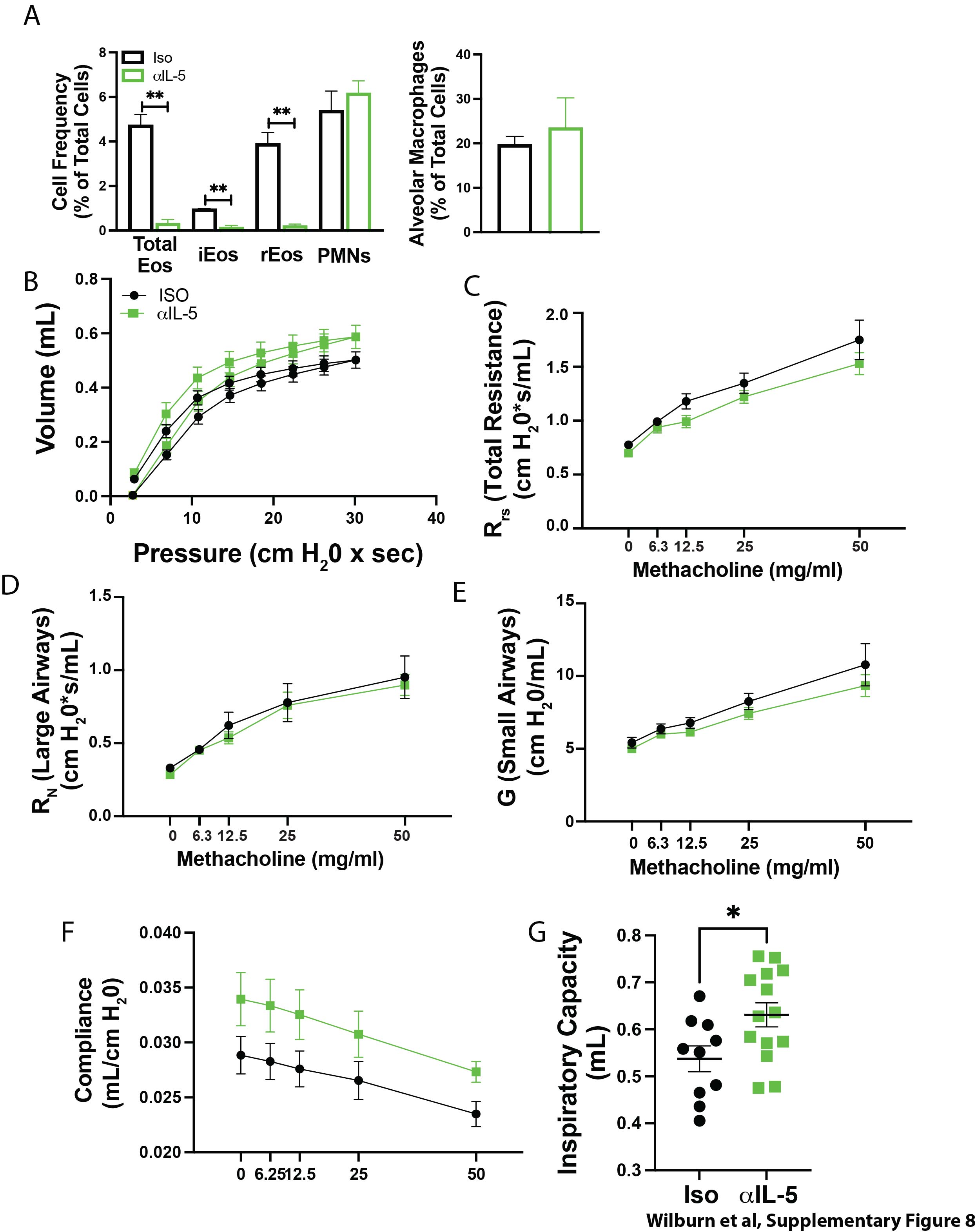

Supplement: Supplementary file 9 [file Image8.jpeg]

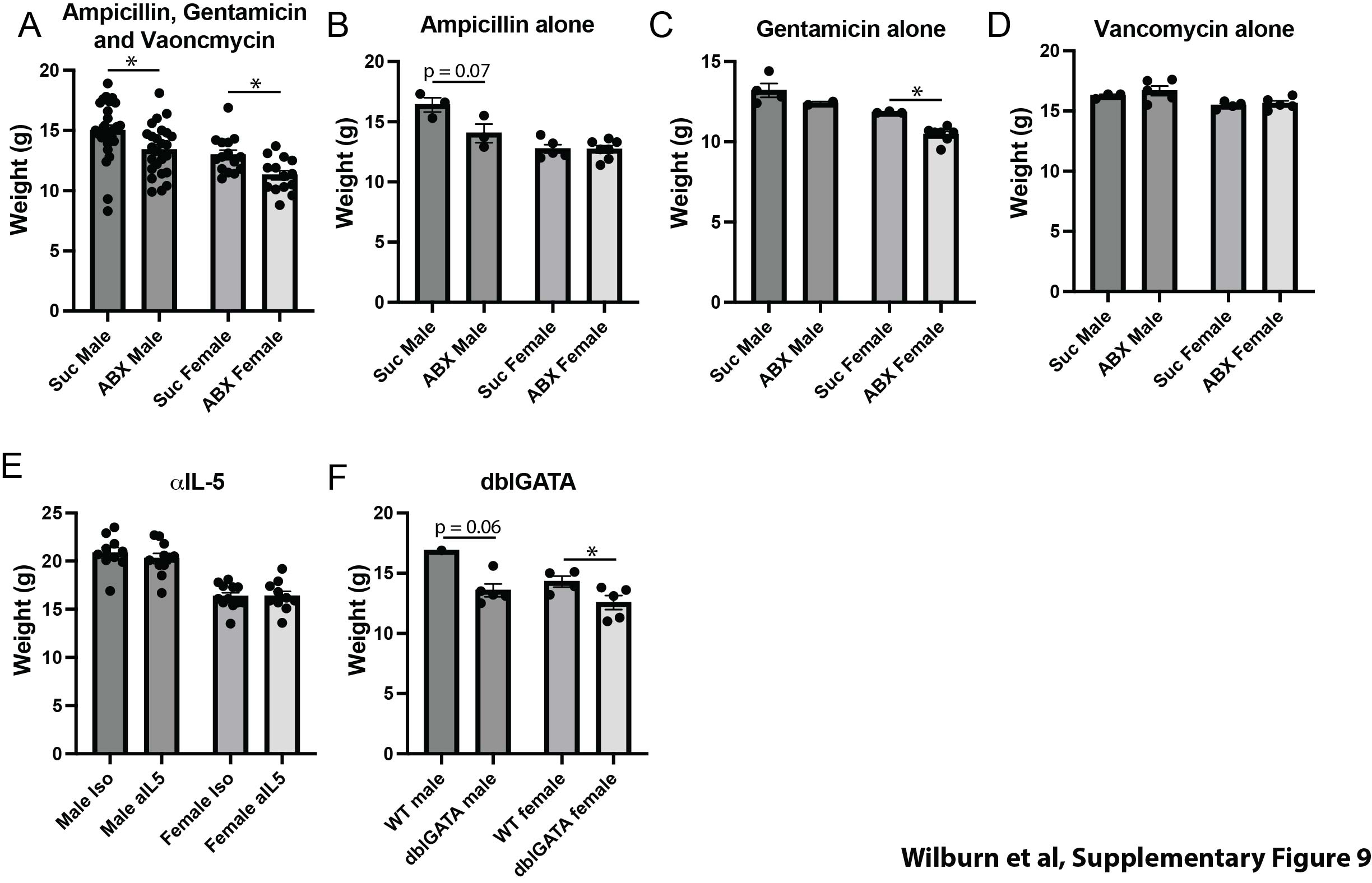

Supplement: Supplementary file 10 [file Image9.jpeg]

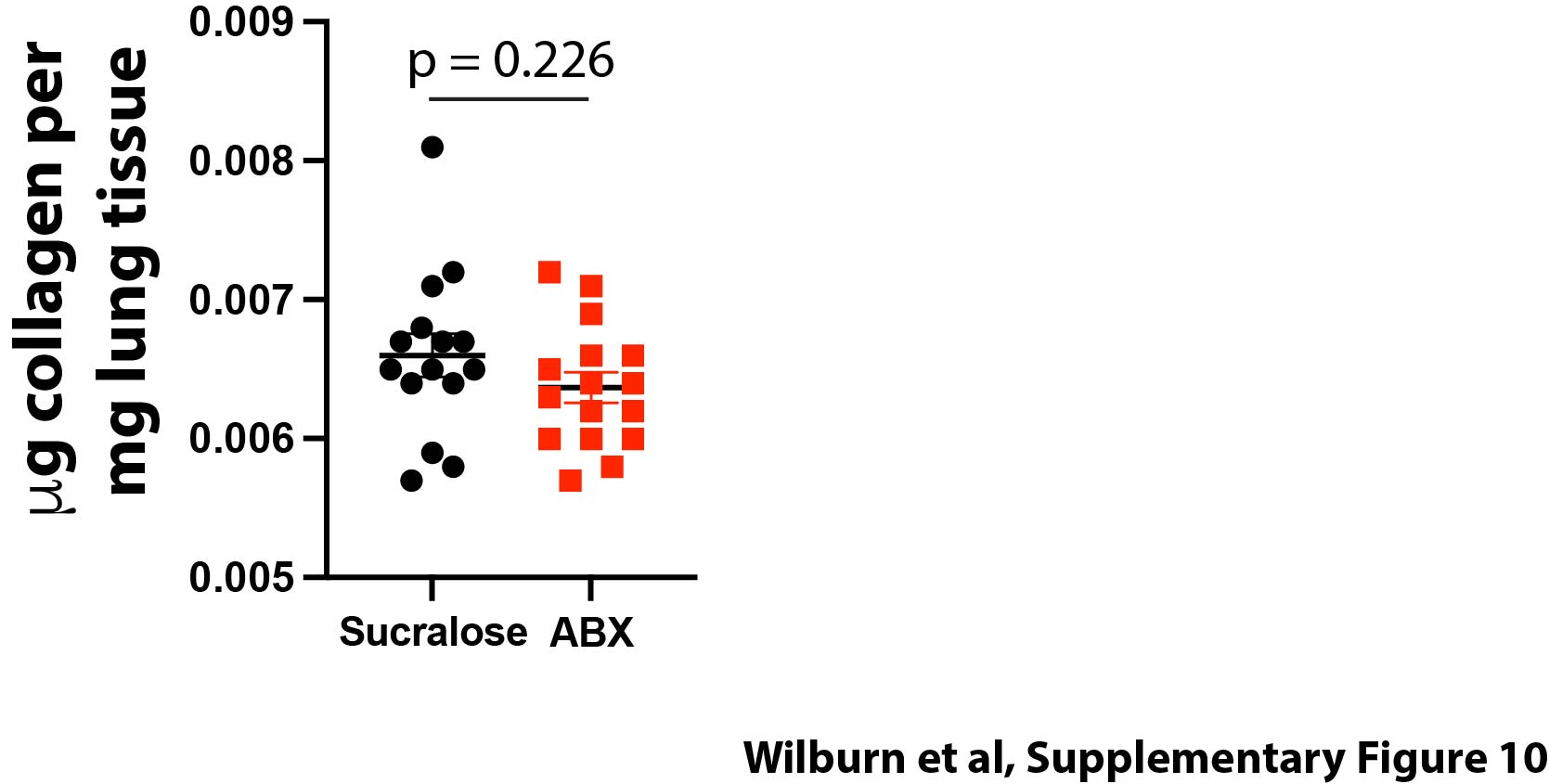

Supplement: Supplementary file 11 [file Image10.jpeg]
